# Solar‐Driven Evaporator With “Starburst Turbine” Design Featuring Directional Salt Crystallization, Antibacterial, and Catalytic Multifunctionality for Efficient Water Purification

**DOI:** 10.1002/advs.202406696

**Published:** 2024-09-25

**Authors:** Jiahui Yu, Lele Li, Yuxuan Liu, Jie Wen, Shu Liu, Jiye Li, Junyi Ning, Changxiang Shao, Tao Wu, Bing Liu

**Affiliations:** ^1^ Department of Stomatology Shandong Provincial Hospital Affiliated to Shandong First Medical University Jinan 250021 China; ^2^ Medical Science and Technology Innovation Center School of Stomatology Shandong First Medical University & Shandong Academy of Medical Sciences Jinan 250117 China; ^3^ Nottingham Ningbo China Beacons of Excellence Research and Innovation Institute University of Nottingham Ningbo China Ningbo 315100 China; ^4^ Department of Chemical and Environmental Engineering The University of Nottingham Ningbo China Ningbo 315100 China

**Keywords:** directional salt crystallization, multifunctional, shape design, solar‐driven evaporator

## Abstract

Facing the global challenge of water scarcity, solar‐driven desalination is considered a sustainable technology for obtaining freshwater from seawater. However, issues such as uncontrolled salt crystallization and bacterial contamination limit its efficiency and practicality. This study proposes an innovative solar‐driven evaporator designed to address these challenges using optimized shape design and advanced photothermal materials. Based on finite element analyses, cylindrical evaporators with a “Starburst Turbine” shape are designed and fabricated, achieving directional salt crystallization and a record‐breaking water collection rate of 3.56 kg m^−2^ h^−1^ and an evaporation rate of 4.57 kg m^−2^ h^−1^ under one sun illumination. During continuous 60‐h illumination tests, the evaporator maintained a stable evaporation rate, attributed to its excellent directional salt crystallization capability. Additionally, the evaporator demonstrates superior photodynamic antibacterial performance and photocatalytic degradation of organic pollutants. Under one sun illumination for 1 h, it achieves 100% sterilization of *S. aureus* and *E. coli*, and a 95.4% degradation of methylene blue (MB), demonstrating its potential to purify various wastewater types. These findings underscore the significant scientific and practical value of integrating antibacterial and photocatalytic functions into solar water purification materials, providing a sustainable solution to global water scarcity challenges and environmental protection.

## Graphical abstract

This study introduces an innovative solar‐driven evaporator with a “Starburst Turbine” design, achieving efficient water purification by optimizing salt crystallization and integrating advanced photothermal materials. It achieves high water collection (3.56 kg m^−2^ h^−1^) and evaporation rates (4.57 kg m^−2^ h^−1^), offers superior antibacterial and photocatalytic capabilities, and provides a sustainable solution for freshwater production and environmental protection.

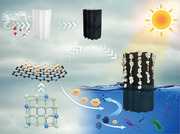

## 1 Introduction

Amid increasing industrialization and human activities, water quality degradation is worsening, and freshwater resources are becoming scarcer.^[^ ^1^ ^]^ Given the complex and variable pollutants and bacteria in water bodies, reverse osmosis (RO) technology is extensively employed to effectively remove organic pollutants and bacteria and to efficiently desalinate seawater.^[^ ^2^ ^]^ This technology not only facilitates the desalination of seawater but also effectively eliminates harmful components from the water. However, RO technology presents significant environmental challenges, such as high energy consumption and the generation of dense brine. Consequently, solar‐powered water purification technology has attracted widespread attention in recent years due to its ability to operate without additional energy input, achieve zero liquid discharge, and recover salts.^[^ ^3^, ^4^ ^]^

One major challenge in applying solar water purification technology to seawater is the localized crystallization of salts.^[^ ^5^ ^]^ During prolonged evaporation processes, salt crystallization on the surface of evaporators forms a salt layer, which impedes the absorption of light and the escape of water vapor, leading to reduced evaporation efficiency.^[^ ^6^ ^]^ Therefore, controlling salt crystallization through evaporator shape design is crucial.^[^ ^7^ ^]^ Researchers have developed conical hydrogel materials that directional salt crystallization primarily to the edges of the cones, preventing deposition on the photothermal material surfaces and thereby ensuring the efficient and stable operation of the evaporator. This evaporator achieves an average evaporation rate of 2.22 kg m^−2^ h^−1^ in seawater over seven days. Other evaporator designs, including umbrella‐shaped,^[^ ^8^ ^]^ disc‐shaped,^[^ ^9^ ^]^ funnel‐shaped^[^ ^10^ ^]^ and microneedle^[^ ^11^ ^]^ configurations, also achieve localized salt crystallization, thus preserving the performance of the surface photothermal materials. Their seawater evaporation performance ranges from 1.17 to 2.8 kg m^−2^ h^−1^. It can be observed that although these shape designs are ingenious, due to limitations in evaporation area and water vapor dispersal, the efficiency of seawater desalination remains low. Therefore, developing an evaporator that both achieves localized salt deposition and high seawater desalination efficiency is of significant importance.

Another challenge facing the practical application of solar water purification technology is the presence of bacteria and contaminants in complex water bodies. During the evaporation process, the warm surrounding environment may promote microbial growth, significantly affecting the lifespan of the evaporator.^[^ ^12^, ^13^ ^]^ Furthermore, the formation of biofilms can cover the surface of photothermal materials, hindering light absorption and water evaporation, thereby reducing the efficiency of solar evaporators.^[^ ^12^ ^]^ Additionally, excessive bacterial growth on the surface of the device could lead to bacterial contamination of the desalinated water, potentially affecting the safety and quality of the produced water.^[^ ^14^ ^]^ Current research employs hydroxyapatite as a carrier to immobilize palladium nanoparticles as catalytic and photothermal materials, achieving an evaporation rate of 1.47 kg m^−2^ h^−1^ and a 100% bacteria removal rate.^[^ ^14^ ^]^ However, the high cost of palladium nanoparticles poses a major barrier to practical application. Recently, reduced graphene oxide (RGO) and polypyrrole (PPy) composite aerogels have also been used to construct antibacterial evaporators;^[^ ^15^ ^]^ however, the preparation process for reduced graphene oxide is complex and similarly expensive. Additionally, the removal of organic contaminants from wastewater remains a challenge.^[^ ^16^, ^17^ ^]^ Photocatalytic technology can effectively degrade organic pollutants in water, thus improving water quality.^[^ ^18^, ^19^ ^]^ Therefore, researching solar seawater purification materials with both antibacterial and photocatalytic capabilities^[^ ^20^ ^]^ is of significant scientific and practical value in addressing global water resource issues and promoting sustainable development and environmental protection.

In this paper, we utilize finite element analysis in simulations and material fabrication in experiments to optimize the external shape and photothermal layer materials of the evaporator. The fabricated “starburst turbine” shaped evaporator not only achieves directed salt crystallization but also consistently and efficiently completes solar‐driven water purification and sterilization processes, while maintaining a high rate of seawater evaporation (4.57 kg m^−2^ h^−1^) and collection (3.56 kg m^−2^ h^−1^). These remarkable outcomes are attributed to the shape design of the evaporator and the modulation of internal structures such as nitrogen doping and oxygen vacancies in the photothermal layer material. Additionally, under 1 h of solar irradiation, the evaporator achieved a remarkable 98.1% removal rate of organic contaminants and 100% sterilization efficacy, demonstrating its outstanding potential for water purification. These results highlight the diverse prospective applications of the evaporator in efficient and sustainable water remediation and purification efforts.

## 2 Results and Discussion

### 2.1 Design of the Evaporator

**Figure 1:**
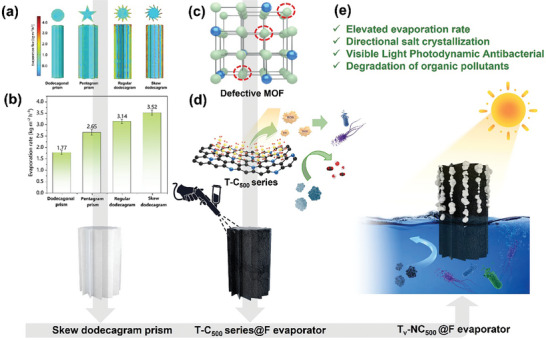
a) Comparison of evaporation fluxes of evaporators with different geometries using finite element simulation; b) Evaporation performance tests of customized MF foams with different shapes (Ti_2_O_3_ as the photothermal layer); c,d) Schematic diagrams of defect MOF precursor and the resulting T‐C_500_ series after calcination; e) Functional schematic diagram of multifunctional solar‐driven seawater evaporator.

To investigate the effect of different evaporator geometries on their evaporation performance, this study employed finite element simulation techniques. As shown in **Figure** **1**a, among the four simulated shapes, the columnar evaporator with a regular dodecagon base exhibits relatively poor performance, which may be attributed to its smaller effective evaporation area. In the designs of columnar evaporators with bases of regular dodecagons, pentagrams, regular dodecagrams, and skew dodecagrams, the effective evaporation area increases progressively, thereby leading to a corresponding increase in evaporation flux. It is noteworthy that the evaporation flux peaked at the sharp corners, as these positions are more conducive to the diffusion of water vapor. This phenomenon indicates that through careful design of the evaporator's shape, localized evaporation of water vapor and collection of salt particles can be achieved. The skew dodecagonal cylindrical evaporators demonstrated the highest evaporation flux. From the top view in Figure 1a, it can be observed that the evaporation flux at the vertices of the skew dodecagonal star is significantly higher than at the center, with the vertices appearing red‐orange in color, resembling a “Starburst Turbine”.

To validate the accuracy of the theoretical simulations, evaporative tests were conducted using the above‐mentioned shapes of Melamine Foam (MF) with Ti_2_O_3_ as the photothermal layer, as shown in Figure 1b. Experimental results confirmed that the “starburst turbine” evaporator exhibited the best seawater desalination performance, consistent with the findings of finite element analysis. Based on these discoveries, the “starburst turbine” shape was selected for further investigation in this study.

As illustrated in Figure 1c,d, in the modification process of the photothermal material, we employed defect nitrogen‐doped titanium‐based metal‐organic framework (Ti‐MOF) material as the precursor. Upon calcination, nitrogen‐doped carbon‐loaded titanium oxide material (T‐C_500_ series) was formed. Through the spray coating technique, this photothermal material was uniformly coated onto the surface of the “Starburst Turbine” cylindrical MF, resulting in the fabrication of a series of solar‐driven evaporators. Subsequent studies will demonstrate that evaporators prepared using this method not only exhibit outstanding solar‐driven seawater desalination performance, but also possess multifunctional characteristics, including localized salt production, sterilization, and degradation of organic matter (as depicted in schematic diagram 1(e)). This multifunctional design ensures the applicability of the solar‐driven evaporator to various water qualities, effectively addressing diverse issues in water and efficiently producing fresh water resources.

### 2.2 Characterization of Photothermal Materials

**Figure 2:**
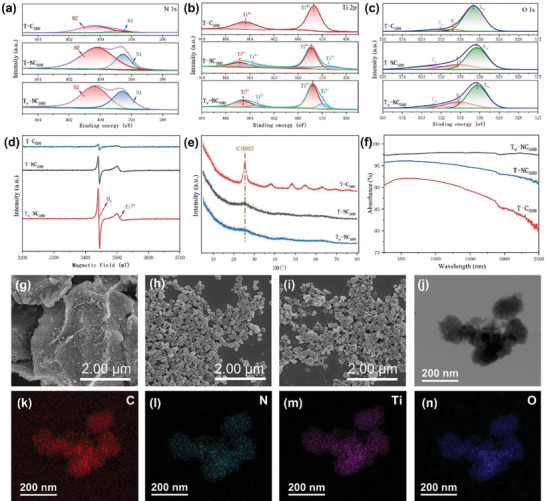
a–c) XPS spectra of N, Ti, and O elements; d) EPR spectra; e) XRD patterns; and f) UV–vis‐NIR absorption spectra of the three photothermal materials. SEM images of g) T‐C_500_, h) T‐NC_500_, and i) T_v_‐NC_500_. HRTEM images and elemental mapping of T_v_‐NC_500_ (j–n).

Due to their extremely high specific surface area, sites amenable to functionalization, and controllable defect structures, MOF materials have become important precursors for the preparation of highly dispersed nanomaterials.^[^ ^21^, ^22^, ^23^ ^]^ In this study, three MOF‐derived photothermal materials were successfully synthesized using a method involving hydrothermal treatment followed by calcination. **Figure** **2** presents the characterization of these three photothermal materials, specifically including carbon‐supported titanium oxide (named T‐C_500_), carbon‐supported nitrogen‐doped titanium oxide (named T‐NC_500_), and carbon‐supported nitrogen‐doped titanium oxide with oxygen vacancies (named T_v_‐NC_500_). The contents of nitrogen doping and oxygen vacancies in these materials were analyzed in detail based on the XPS results. As shown in Figure 2a, the peak intensity of nitrogen in T‐NC_500_ and T_v_‐NC_500_ is significantly higher compared to T‐C_500_, indicating that the nitrogen doping process was successfully completed, thereby significantly improving the hydrophilicity of the materials (see Figure S1, Supporting Information). Compared to T‐C_500_ without nitrogen doping, both T‐NC_500_ and T_v_‐NC_500_ exhibit superior hydrophilicity due to the stronger attraction of nitrogen atoms to water molecules.^[^ ^24^ ^]^ In addition, compared to T‐NC_500_, the material with more oxygen vacancies (T_v_‐NC_500_) shows even better hydrophilicity, as oxygen vacancies enhance the adsorption of water molecules on the material surface.^[^ ^25^ ^]^ The improved hydrophilicity of the photothermal materials can ensure a consistent water supply during the evaporation process, which is expected to increase the water evaporation rate. Among the nitrogen species, the content of pyridinic nitrogen (N1) increased most significantly, suggesting that this nitrogen doping method predominantly increases the amount of pyridinic nitrogen (N1). As shown in **Table** **1**, the N1 content in T_v_‐NC_500_ is the highest, reaching 42.7%, whereas the N1 content in T‐C_500_ is only 17.4%. Theoretical calculations have shown that pyridinic nitrogen (N1), compared to pyrrolic nitrogen (N2), has a greater affinity for water molecules and more effectively weakens the hydrogen bonds between water molecules.^[^ ^24^ ^]^ Figure S3 (Supporting Information) shows the equivalent evaporation enthalpy (H_S_) calculated according to Equation S3 (Supporting Information). The results indicate that T_v_‐NC_500_ has the lowest equivalent evaporation enthalpy, while T‐C_500_ has the highest, which is consistent with the relative content of N1. Since the trend of the evaporation enthalpy is consistent with the trend in the content of pyridinic nitrogen (N1), it is speculated that the main reason for the reduced evaporation enthalpy is the stronger affinity of nitrogen‐doped carbon materials (primarily N1) for water molecules, which weakens the hydrogen bonding between water molecules.^[^ ^24^ ^]^ As a result, less energy is required for the water molecules to transition from the liquid to the gaseous state. This demonstrates that nitrogen doping not only improves the hydrophilicity of photothermal materials but also plays a significant role in reducing the evaporation enthalpy.

**Table 1:** Peak Proportions of Different Elements in T‐C_500_ Series Samples.

	T‐C_500_	T‐NC_500_	T_v_‐NC_500_
N1/(N1+N2)	17.4%	29.8%	42.7%
Ti^3+^/(Ti^4+^+Ti^3+^)	0.0%	9.5%	15.8%
V_o_/(V_o_+C_o_+L_o_)	3.9%	14.2%	22.7%

Figure 2b presents the content of Ti in different valence states, where the peaks at 458.5 eV and 464.3 eV correspond to Ti⁴⁺,^[^ ^26^ ^]^ and the peaks at 457.8–458.0 eV and 463.4–463.6 eV are considered characteristic of Ti^3^⁺.^[^ ^27^ ^]^ It can be seen that the Ti^3^⁺ peaks are almost absent in T‐C_500_, indicating the lack of oxygen vacancies in this material. In T‐NC_500_, a small amount of Ti^3^⁺ peaks can be observed, suggesting that nitrogen doping promotes the formation of a few oxygen vacancies. For the T_v_‐NC_500_ material, the Ti^3^⁺ peaks are more prominent. To further determine the ratio of Ti^3^⁺ to Ti⁴⁺, the ratio was calculated by analyzing the XPS peak areas. As shown in Table 1, the Ti^3^⁺ content in T_v_‐NC_500_ is significantly higher than in T‐NC_500_ (9.5% vs 15.8%), demonstrating a notable increase.

To further confirm the presence of oxygen vacancies, the XPS peaks of oxygen were analyzed. Generally, the peak positions for lattice oxygen (L_o_) and chemisorbed oxygen (C_o_) are in the ranges of 530.0–530.3 eV and 532.1–532.4 eV, respectively. The oxygen vacancy (V_o_) is approximately at 531.2 eV.^[^ ^28^ ^]^ The L_o_ peak corresponds to the O^2^⁻ atoms in the lattice, which are fully coordinated with Ti⁴⁺ in the TiO₂ lattice. The V_o_ is attributed to oxygen vacancies of O^2^⁻ in the matrix, while the C_o_ originates from oxygen or OH species dissociated from H₂O or O₂ in the surrounding environment.^[^ ^28^ ^]^ As shown in Figure 2c, compared to the other two materials, the V_o_ content in T_v_‐NC_500_ is the highest, which is consistent with the XPS results of Ti 2p, collectively indicating the successful preparation of oxygen vacancies in the T_v_‐NC_500_ material.

Figure 2e presents the XRD results of the three materials. The peak at 25° corresponds to the (002) plane of graphite carbon, representing the graphite crystal structure. The C (002) peak shows a sharp spike only in T‐C_500_, while it appears diffuse in T‐NC_500_ and T_v_‐NC_500_. This indicates that the graphite crystallinity is highest in T‐C_500_. In contrast, the formation of atomic doping and vacancies in the other materials hinders the formation of a carbon lattice, potentially leading to the creation of more defect states in the carbon material.

To investigate the light absorption capabilities of different photothermal materials, their UV/Vis‐NIR absorption spectra were measured. As shown in Figure 2f, T_v_‐NC_500_ exhibits broad light absorption across the entire UV/Vis to NIR region, with an absorption efficiency reaching up to 97%. In comparison, the light absorption capabilities of T‐C_500_ and T‐NC_500_ are significantly weaker, particularly T‐C_500_, which shows very low absorption in the near‐infrared region. This result indicates that the light absorption capacity of T_v_‐NC_500_ has been significantly enhanced due to the nitrogen doping and the formation of oxygen vacancies.

Figures 2g‐i respectively show the morphologies of T‐C_500_, T‐NC_500_, and T_v_‐NC_500_. It can be seen that the particle size of T‐C_500_ is significantly larger than those of the other two materials, which is consistent with the higher crystallinity of T‐C_500_ observed in the XRD results. This further indicates that nitrogen doping and the formation of oxygen vacancies affect the particle size and crystallinity of the materials. Figure 2j–n) present the high‐resolution transmission electron microscopy (HRTEM) images and elemental distribution maps of the T_v_‐NC_500_ material. The distributions of nitrogen, carbon, titanium, and oxygen are seen to be very uniform, demonstrating that this preparation method can effectively achieve uniform doping of multiple elements, thereby enhancing the overall performance of the material.

The above characterization results demonstrate that using defective MOFs as precursors allows for effective and precise control of nitrogen doping and oxygen vacancies. The presence of nitrogen doping and oxygen vacancies significantly enhances the light absorption properties and hydrophilicity of the material. Furthermore, this preparation method results in highly dispersed materials, ensuring structural uniformity in the photothermal materials.

### 2.3 Seawater Desalination Performance, Directed Salt Crystallization, and Stability Testing

**Figure 3:**
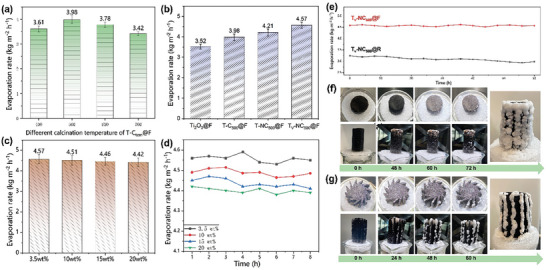
a) Desalination performance of different T‐C_tem_@F evaporators in 3.5 wt% brine; b) Desalination performance of evaporators made from different photothermal materials; c,d) Evaporation performance and 8‐h evaporation test of the T_v_‐NC_500_@F evaporator in brine solutions of varying concentrations; e) Stability test of the evaporation performance of T_v_‐NC_500_@F and T_v_‐NC_500_@R evaporators in 3.5 wt% brine over 60 h; f,g) Distribution of salt crystallization on the T_v_‐NC_500_@F evaporator and the T_v_‐NC_500_@R evaporator.

To determine the optimal calcination temperature for the T‐C_tem_ series materials, a series of T‐C_tem_@F evaporators were prepared, and their desalination performance was evaluated, as shown in **Figure** **3**a. After completing the tests on the height of the evaporators (as shown in Figure S6, Supporting Information), the height of all evaporators was set to 4 cm. Among these, the desalination rate of the T‐C_500_@F evaporator reached 3.98 kg m⁻^2^ h⁻¹, demonstrating superior evaporative performance. Therefore, the T‐C_500_ material was selected as the basis for further optimization. Figure 3b compares the evaporation rates of the optimized T‐NC_500_ and T_v_‐NC_500_ evaporators, which were prepared as photothermal layers, with those of evaporators prepared using T‐C_500_ and Ti_2_O_3_.

The results indicate that the T_v_‐NC_500_@F evaporator exhibited the highest evaporation rate, reaching 4.57 kg m⁻^2^ h⁻¹, followed by the T‐NC_500_ evaporator at 4.21 kg m⁻^2^ h⁻¹. This suggests that both nitrogen doping and oxygen vacancy regulation in the photothermal materials can enhance the performance of the evaporator to a certain extent, which is closely related to the enhancement of light absorption capability and improvement of hydrophilicity induced by nitrogen doping and oxygen vacancies (as shown in Figure 2f; Figure S1, Supporting Information). Furthermore, the mechanical properties of the evaporator are also key indicators that affect its practical application.^[^ ^29^, ^30^ ^]^ Therefore, the mechanical properties of the T_v_‐NC_500_@F evaporator were tested. As shown in Figure S5 (Supporting Information), the stiffness and strength of the evaporator significantly increased with strain, indicating good elastoplastic characteristics and high compressive resistance. This demonstrates that the T_v_‐NC_500_@F evaporator has high mechanical stability during practical use.

Solar‐to‐vapor conversion efficiencies were calculated for the optimized evaporators in Figure 3b to explore their light utilization capabilities. After considering dark evaporation, the solar‐to‐vapor conversion efficiencies were determined to be 8.15, 82.4, 78.7, and 84.5% for MF foam, T‐C_500_@F, T‐NC_500_@F, and T_v_‐C_500_@F, respectively, based on their equivalent enthalpies^[^ ^31^ ^]^ (as illustrated in Figure S3, Supporting Information). This indicates that the three evaporators fabricated using three prepared photothermal materials all exhibit high solar‐to‐vapor conversion efficiencies, each exceeding 75%. This demonstrates that these evaporators possess excellent photothermal conversion capabilities.

Subsequently, the evaporator with the best evaporation performance, T_v_‐NC_500_@F, was selected for desalination tests with different concentrations of brine, as shown in Figure 3c. Even in brine concentrations as high as 20 wt%, the T_v_‐NC_500_@F evaporator still demonstrated excellent evaporation capability, reaching 4.42 kg m⁻^2^ h⁻¹, highlighting its outstanding potential in high‐concentration brine environments. Moreover, under 8 h of continuous illumination, the evaporation rate of this evaporator did not exhibit a significant decrease, as illustrated in Figure 3d.

Furthermore, Figures 3e–g compare the long‐term stability and directional salt crystallization ability of T_v_‐NC_500_@R and T_v_‐NC_500_@F evaporators under continuous illumination in 3.5 wt% brine. Specifically, T_v_‐NC_500_@R refers to an evaporator made by spraying the T_v_‐NC_500_ material on a regular dodecagonal prism. The evaporation rate of the T_v_‐NC_500_@F evaporator is significantly better than that of the T_v_‐NC_500_@R evaporator, consistent with the finite element analysis results in Figure 1a, indicating that the shape of the evaporator has a significant impact on its evaporation performance. During the 60‐h stability test, the T_v_‐NC_500_@F evaporator maintained a stable evaporation performance of 4.5–4.6 kg m⁻^2^ h⁻¹, without a decrease due to salt crystallization. This is because all the salt crystallized on the edges of the skew dodecagram column shape (as shown in Figure 3g), preventing crystallization on the top and sides of the evaporator. As a result, salt crystallization did not affect light absorption, which is also consistent with the finite element analysis results. In contrast, the evaporation performance of the T_v_‐NC_500_@R evaporator was much lower. During the 60‐h stability test, the evaporation performance of the T_v_‐NC_500_@R evaporator gradually decreased from the initial 3.24 kg m⁻^2^ h⁻¹ to ≈2.98 kg m⁻^2^ h⁻¹. The decline was not significant, it was due to the relatively low evaporation rate, thus salt crystallization had not yet accumulated on the evaporator surface. In subsequent tests (as shown in Figure 3f), salt crystallization covered the entire surface of the T_v_‐NC_500_@R evaporator, inevitably impairing its evaporation performance, resulting in a significant reduction of the evaporation rate to 2.04 kg m⁻^2^ h⁻¹. In contrast, all the salt crystals on the T_v_‐NC_500_@F evaporator concentrated on the edges and could freely fall off, achieving a stable evaporation rate of 4.4 kg m⁻^2^ h⁻¹ and a salt collection rate of 0.10 kg m⁻^2^ h⁻¹. This provides new insights for the stable operation and reliable salt production of evaporators.

### 2.4 Organic Degradation Capability, Antibacterial Activity, and Mechanism Analysis

**Figure 4:**
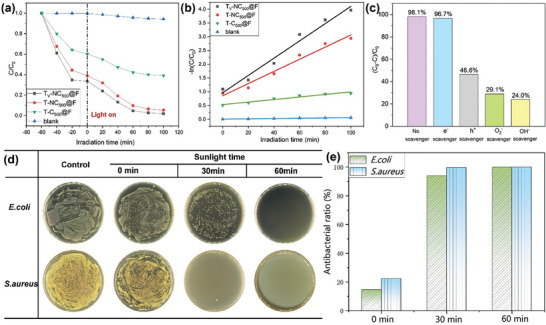
a) Photocatalytic degradation of MB by the prepared samples over time; b) Kinetic fit for the degradation of MB by the prepared samples; c) Photocatalytic degradation of MB by T_v_‐NC_500_@F in the presence of different scavengers under simulated solar light; d,e) Photodynamic antibacterial performance of the T_v_‐NC_500_@F evaporator.

To investigate the ability of different evaporators to degrade water containing organic pollutants, methylene blue (MB) solution was subjected to photodegradation to evaluate its photoactivity (**Figure** **4**a). Among the samples, T_v_‐NC_500_@F, which has both nitrogen doping and oxygen vacancies, exhibited the best photocatalytic degradation ability for MB solution, achieving a removal rate of 95.4% after 60 min of irradiation and 98.1% after 100 min of irradiation. In comparison, the T‐NC_500_@F sample, which lacks customized oxygen vacancies, showed slightly weaker catalytic degradation ability with a removal rate of 94.7%. The presence of nitrogen doping had a significant impact on the removal rate of MB; the T‐C_500_@F sample, which lacks nitrogen doping, achieved only a 61.0% removal rate after 100 min of irradiation.

The pseudo‐first‐order‐model is employed to evaluate the photocatalysis kinetics Equation (1):^[^ ^32^ ^]^

(1) $$\ln\left(\frac{C}{C_{0}}\right)=-kt$$

where *C* is the MB concentration, *C_0_* is the initial MB concentration, *t* is the irradiation time, and *k* is the pseudo‐first‐order constant (min^−1^). As shown in Figure 4b, the order of the photodegradation rates for all samples is as follows: T_v_‐NC_500_@F (0.03187 min^−1^) > T‐NC_500_@F (0.02207 min^−1^) > T‐C_500_@F (0.0046 min^−1^), confirming that the T_v_‐NC_500_@F sample, which contains nitrogen doping and oxygen vacancies, exhibits the best photocatalytic performance among all the samples.

To understand the role of active radicals in the photocatalytic process, radical scavenging experiments were performed on the T_v_‐NC_500_@F sample. As shown in Figure 4c, compared to the control group (no scavenger added), the degradation efficiency of MB after 100 min of irradiation decreased by only 1.4% when an electron (e⁻) scavenger (silver nitrate) was added. The effect of adding a hole (h⁺) scavenger (potassium oxalate) was more significant, reducing the degradation efficiency of MB to 46.6%. Even more pronounced effects were observed with the hydroxyl radical (·OH) scavenger (isopropanol) and the superoxide anion radical (O_2_·^−^) scavenger (p‐benzoquinone), which greatly suppressed the photocatalytic efficiency of T_v_‐NC_500_@F, reducing the degradation efficiency to 29.1% and 24.0%, respectively. These results indicate that e^−^ plays almost no role in the photocatalytic degradation process, h^+^ plays a minor role, while O_2_·^−^and ·OH are the main active species in the photocatalytic process. This suggests that the T_v_‐NC_500_@F material can generate a large amount of reactive oxygen species under illumination. To study its photocatalytic stability, the recycling photodegradation experiments of the T_v_‐NC_500_@F material were conducted, as shown in Figure S4 (Supporting Information). The results show that the T_v_‐NC_500_@F material exhibits excellent stability over 5 cycles of photodegradation, ensuring its reusability.

The active oxygen species (·OH and O_2_^−^) generated under illumination are expected to provide antibacterial capability, potentially reducing the risk of evaporator contamination due to the increase in bacterial numbers in natural water sources during long‐term use. Therefore, the antibacterial ability of the T_v_‐NC_500_@F evaporator was investigated using Staphylococcus aureus (*S. aureus*) and Escherichia coli (*E. coli*) as experimental models, with the number of colonies on agar plates in the control group as a reference. As shown in Figure 4d,e, the T_v_‐NC_500_@F evaporator exhibited significant antibacterial effects against both *S. aureus* and *E. coli*. The antibacterial rate increased progressively with prolonged illumination time. Since T_v_‐NC_500_@F can generate reactive oxygen species (ROS) under visible light during photothermal conversion, its antibacterial ability is likely due to the combined effects of temperature and ROS. Figure S8 (Supporting Information) analyzes the antibacterial effect of a control sample that was heated to the evaporator's surface temperature (47.2 °C) without providing ROS. The results indicate that merely raising the temperature does not achieve a good antibacterial effect. Therefore, the ROS generated by the material under illumination is the primary reason for the excellent antibacterial ability of the T_v_‐NC_500_@F evaporator, while the increase in temperature under illumination also aids in disinfection. After 30 min of illumination, the antibacterial rates against *E. coli* and *S. aureus* reached 94.0 and 99.7%, respectively. After 60 min of illumination, the T_v_‐NC_500_@F evaporator achieved an antibacterial rate of 100% against both *E. coli* and *S. aureus*, demonstrating excellent antibacterial performance.

Additionally, to test the stability of the antibacterial ability of the T_v_‐NC_500_@F evaporator, the antibacterial performance of the photothermal material was evaluated after 5 days of testing (as shown in Figure S10, Supporting Information). It is evident that after 30 min of illumination, the number of bacteria significantly decreased, and after 60 min, a 100% sterilization effect was achieved. This result is consistent with the antibacterial performance of the unused T_v_‐NC_500_@F (as shown in Figure 4d), indicating good temporal stability of the photothermal material. It can maintain its excellent antibacterial ability even after prolonged operation, which is crucial for the continuous stable operation and practical application of the evaporator.

**Figure 5:**
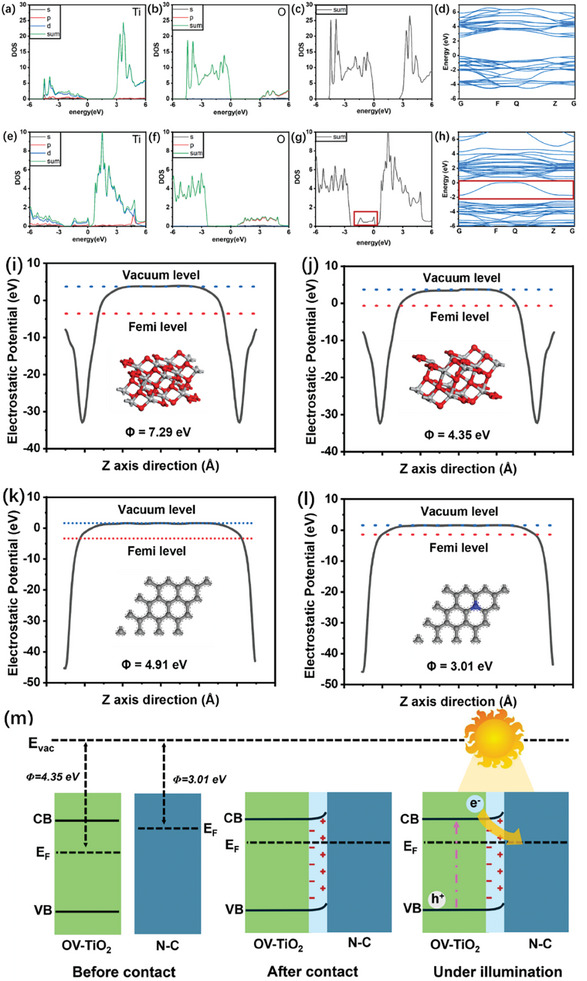
a–d) Density of States (DOS) and band structure diagrams of TiO₂; e–h) DOS and band structure diagrams of O_v_‐TiO₂; i) Work function of TiO₂; j) Work function of O_v_‐TiO₂; k) Work function of carbon; l) Work function of N‐doped carbon (N‐C); m) Charge transfer pathway in O_v_‐TiO₂/N‐C.

To gain a deeper understanding of the impact of oxygen vacancies (O_v_) and nitrogen doping on the structure and electron transfer of the T_v_‐NC_500_@F material, we conducted density functional theory (DFT) calculations. A molecular structure model of T_v_‐NC_500_ was established using TiO₂ with oxygen vacancies and nitrogen‐doped graphene. To further explore the role of O_v_ in the electronic and optical properties of the O_v_‐TiO₂ structure, **Figures** **5**a–h) show the density of states (DOS) and band structure diagrams of pure TiO₂ and O_v_‐TiO₂, respectively. As illustrated in Figure 5, the valence band maximum (VBM) is primarily composed of O‐2p orbitals, while the conduction band minimum (CBM) is mainly derived from Ti orbitals. Compared to pristine TiO₂, O_v_‐TiO₂ exhibits two sets of O_v_‐induced states: one set overlaps with the CB, derived from Ti orbitals, thereby reducing the bandgap of O_v_‐TiO₂; the other set is considered intermediate energy levels, also derived from Ti orbitals, enhancing the tail absorption.^[^ ^33^ ^]^

Furthermore, the direction of charge transfer at the heterojunction interface of compound semiconductors is reflected by the work function (Φ), which is defined as Φ = E_vac_ – EF, where E_vac_ and EF represent the vacuum energy level and the Fermi energy level, respectively.^[^ ^34^ ^]^ DFT calculations revealed that the work functions of pure TiO₂ and graphite carbon are 7.29 and 4.91 eV, respectively (as shown in Figures 5i,k). Figures 5j,l indicate that the presence of oxygen vacancies reduces the work function of TiO₂ to 4.35 eV, while nitrogen doping decreases the work function of graphite carbon to 3.01 eV. This suggests that electron transfer between the surfaces of O_v_‐TiO₂ and N‐C becomes more feasible. Figure 5m shows the band structures of O_v_‐TiO₂ and N‐C and illustrates the charge transfer process under different conditions after forming the composite material O_v_‐TiO₂/N‐C. The results indicate that the Fermi level of O_v_‐TiO₂ is lower than that of N‐C, implying that when they come into contact, electrons will flow from N‐C to O_v_‐TiO₂ until they reach the same EF level. Simultaneously, an electron accumulation layer and depletion layer form near the interface between O_v_‐TiO₂ and N‐C, creating an internal electric field (IEF) directed from N‐C to O_v_‐TiO₂ at the contact interface. This facilitates the separation and transfer of photoinduced charge carriers in the O_v_‐TiO₂/N‐C structure, leveraging the excellent conductivity of N‐C.^[^ ^35^, ^36^ ^]^ When light irradiates the surface of O_v_‐TiO₂/N‐C, photoexcited electrons are driven by the IEF of the heterojunction to transfer from the CB of TiO₂ to N‐C, leaving holes in the VB. Due to the repulsive force of the electric field at the interface, the holes cannot effectively transfer. Consequently, the spatial separation of photoinduced electrons and holes effectively prevents their recombination, which is a primary reason for the excellent photocatalytic performance of the T_v_‐NC_500_@F material.

Additionally, the analysis from DFT calculations elucidates the key mechanism behind the photodynamic antibacterial capability of the T_v_‐NC_500_@F material shown in Figure 4. The effective separation of photogenerated electrons and holes is a prerequisite for the generation of reactive oxygen species (ROS). Specifically, photogenerated electrons react with dissolved oxygen (O₂) to produce superoxide anions (O₂·⁻), while photogenerated holes can oxidize water (H₂O) or hydroxide ions (OH⁻) to generate hydroxyl radicals (·OH). Therefore, the separation of photogenerated electrons and holes is crucial for the generation of ROS, which significantly enhances the photodynamic antibacterial capability of the T_v_‐NC_500_@F material.

### 2.5 Outdoor Seawater Evaporation and Collection and Purification of Various Water Sources

**Figure 6:**
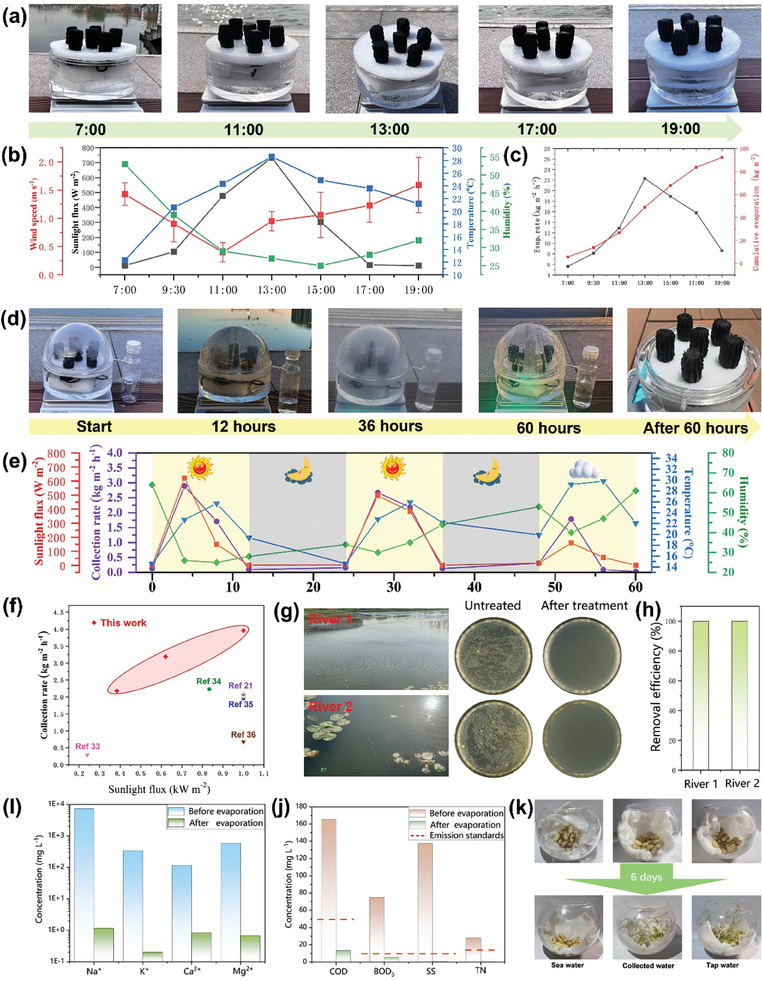
a) Photograph of the T_v_‐NC_500_@F evaporator during a 12‐h outdoor evaporation performance test with 3.5 wt% brine; b,c) Outdoor environmental parameters and evaporation rate of the T_v_‐NC_500_@F evaporator; d) Photograph of the T_v_‐NC_500_@F evaporator during a 60‐h outdoor water collection test; e) Outdoor environmental parameters and water collection rate during the test; f) Comparison of the water collection capacity of the T_v_‐NC_500_@F evaporator with literature data; g) Agar plates showing bacterial cultures before and after treatment of river water by the T_v_‐NC_500_@F evaporator; h) Bacteria removal efficiency for two river water samples; Purification capability of the T_v_‐NC_500_@F evaporator for i) seawater and j) urban wastewater; k) Use of purified water for bean sprout cultivation.

Under real environmental conditions, a seawater desalination experiment was conducted using an array of six T_v_‐NC_500_@F evaporators. The design of the evaporation device^[^ ^37^ ^]^ is shown in Figure S13 (Supporting Information). For more details, see the Supporting Information. During the 12‐h evaporation, no salt accumulation was observed (as shown in **Figure** **6**a). Figure 6b records the environmental data from 7:00 AM to 7:00 PM, including temperature, wind speed, light intensity, and humidity. At 13.00 PM, both the ambient temperature and solar radiation reached their peak values for the day. Correspondingly, the evaporation rate also reached its maximum at this time, as shown in Figure 6c. As illustrated in Figure 6c, the total evaporation for the day reached 92.2 kg m⁻^2^, highlighting the superior evaporation performance and significant practical application potential of the system. Furthermore, to ensure the safety of the evaporator, COD, UV–vis, and ICP‐MS were used to detect whether the water soaked with T_v_‐NC_500_@F and T‐NC_500_@F was contaminated. As shown in Figure S12 (Supporting Information), the absorbance of pure water is not different from that of the water samples after being soaked with T_v_‐NC_500_@F and T‐NC_500_@F for five days. Additionally, the COD and titanium concentrations in the water samples remained at very low levels, fully confirming the safety of the evaporator.

In practical applications, the water collection capacity of the evaporator is particularly important. Figure 6d shows photos of the T_v_‐NC_500_@F evaporator during a 60‐h outdoor water collection test. During the 60‐h day‐night alternating evaporation process, no salt accumulation was observed on the evaporator surface, which contrasts with the salt accumulation observed after 60‐h of continuous illumination (as shown in Figure 3e). This difference is due to the salt returning to the water through the porous foam during the night when there is no illumination. The 60‐h water collection process produced a substantial amount of fresh water, with a total yield of 178 ml. Figure 6e presents the environmental variables and water collection data during the day‐night alternating water collection process. Under sunny conditions during the first two days, the water collection rate remained relatively stable (as indicated by the purple line in the figure). However, on the third day, when it was cloudy, the water collection rate decreased significantly, indicating that sunlight has a considerable impact on the evaporation and collection rate. Figure 6f compares the water collection rates of the T_v_‐NC_500_@F evaporator under different light intensities with data from the literature.^[^ ^22^, ^38^, ^39^, ^40^, ^41^ ^]^ The T_v_‐NC_500_@F evaporator demonstrates excellent water collection capabilities under all light intensity conditions. Notably, at a solar intensity of one sun, its water collection rate reaches 3.56 kg m⁻^2^ h⁻¹, making it the best‐reported water collection rate to date.

Figure 6g,f shows the effectiveness of the T_v_‐NC_500_@F evaporator in purifying river water, with bacteria completely removed from the evaporated water, achieving a 100% bacteria removal rate. Additionally, the T_v_‐NC_500_@F evaporator can also be used to purify seawater and urban wastewater (Figure 6l,j). As shown in Figure 6l, after desalination, the concentrations of four typical ions (Na⁺, K⁺, Ca^2^⁺, and Mg^2^⁺) in real seawater significantly decreased to 1.15, 0.2, 0.82, and 0.67 mg L⁻¹, respectively, meeting the World Health Organization (WHO) recommended standard for drinking water.^[^ ^42^ ^]^ Figure 6j demonstrates the T_v_‐NC_500_@F evaporator's ability to purify urban wastewater, with the levels of chemical oxygen demand (COD), biochemical oxygen demand (BOD_5_), suspended solids (SS), and total nitrogen (TN) all reaching the standards for wastewater discharge, highlighting the T_v_‐NC_500_@F evaporator's capability to treat various types of water sources. Figure 6k illustrates the use of purified water for sprouting bean seeds. The seeds soaked in unpurified seawater did not germinate, while those soaked in purified water and tap water germinated well, demonstrating the usability of the purified water.

## 3 Conclusion

In this study, we developed a novel solar‐driven evaporator featuring a “Starburst Turbine” design to address the significant challenges of uncontrolled salt crystallization and bacterial contamination in water purification systems. By optimizing the evaporator's shape and employing advanced photothermal materials, we achieved localized salt crystallization, thus maintaining the efficiency of the photothermal surfaces. Finite element analysis guided the design of a cylindrical evaporator with a “Starburst Turbine” shape, which was fabricated to achieve an impressive seawater evaporation rate of 4.57 kg m^−2^ h^−1^ and a freshwater collection rate of 3.56 kg m^−2^ h^−1^ under one sun illumination. The stability of the evaporator was demonstrated in continuous 60‐h illumination tests, showing no decline in evaporation rate due to the efficient targeted salt collection. Furthermore, the evaporator exhibited excellent multifunctionality, including superior photodynamic antibacterial performance and photocatalytic degradation of organic pollutants. It achieved 100% sterilization of *S. aureus* and *E. coli* and a 95.4% degradation rate of methylene blue within 1 h of solar illumination, highlighting its potential for comprehensive water purification applications.

These findings underscore the significant scientific and practical value of integrating antibacterial and photocatalytic functions into solar water purification materials. This approach offers a sustainable and efficient solution to global water scarcity challenges and environmental protection, paving the way for the development of advanced materials in water treatment technologies.

## 4 Experimental Section

### Preparation Details**—**Preparation of T‐C_tem_

0.03 mol of terephthalic acid was completely dissolved in 125 mL of DMF (N,N‐dimethylformamide, Sinopharm) and anhydrous methanol (13.5 mL, Sinopharm). Then, 2.6 mL of TBT (tetrabutyl titanate, McLean) was quickly added and stirred for 30 min. The solution was placed in a polytetrafluoroethylene‐sealed autoclave and reacted at 150 °C for 24 h. After the reaction, the product was washed three times with anhydrous methanol and dried under vacuum at 80 °C to obtain MIL‐125. Finally, MIL‐125 was calcined at 400, 500, 600, and 700 °C for 5 h in a nitrogen atmosphere to obtain T‐C_tem_ (“tem” represents calcination temperature). After desalination testing, the results (Figure 3a) reveal that the material prepared by calcination at 500 °C exhibits the highest evaporation performance when used as the photothermal material. Consequently, T‐C_500_ was selected as the target for further optimization in subsequent experiments.

### Preparation Details**—**Preparation of T‐NC_500_ and Tv‐NC_500_

In further optimization processes, for the preparation of T‐NC_500_, the 0.03 mol of terephthalic acid ligand used in T‐C_500_ was replaced with 0.03 mol of 2‐NH₂‐terephthalic acid. For the preparation of T_v_‐NC_500_, the same amount of terephthalic acid ligand was substituted with a mixture of 0.027 mol of 2‐NH₂‐terephthalic acid and 0.003 mol of terephthalic acid. All other steps remained identical to those used in the preparation of T‐C_500_.

### Preparation Details**—**Preparation of T‐C_500_@F, T‐NC_500_@F, T_v_‐NC_500_@F and T_v_‐NC_500_@R

The spraying process was carried out using a spray gun connected to a gas cylinder. In the nomenclature of the evaporators, *F* and *R* respectively represent the evaporators with a “Starburst Turbine” shape and with a regular dodecagon as the base, respectively. T‐C_500_, T‐NC_500_, and T_v_‐NC_500_ represent different photothermal materials. The water‐absorbing cotton rope is inserted from the center of the bottom of the cylindrical evaporator to ensure a consistent water supply (Figure S6, Supporting Information). Further details regarding spraying techniques and the cost of the evaporator are provided in the Supporting Information.

### Solar Desalination Experiment

The performance evaluation of the evaporator was conducted employing a solar simulator (CEL‐S500‐T5, CEAULIGHT, China). Before commencing the experimental protocol, the intensity of light incident on the evaporator surface was quantified using a light power meter (CEL‐NP2000‐2A, CEAULIGHT, China). To ensure experimental precision, a five‐spot measurement strategy was employed for calibrating the light intensity. During the experiment, a cup was used as a container to hold various types of water samples to be purified, including different concentrations of saline solutions (3.5, 10, 15, and 20 wt%), simulated seawater containing various ions, urban wastewater, and river water from the school. The photothermal device was placed inside the cup, and a white EPS foam board was used to cover the exposed water surface to prevent direct evaporation of water vapor. The evaporation rate was determined by real‐time measurement of water weight loss using a high‐precision electronic mass balance (FA2004, SUNNY HENGPING, China) with an accuracy of up to 0.0001 g. Simultaneously, the surface temperature of the evaporator was monitored using an advanced infrared camera (FOTRIC, 628C‐L25, China). Throughout the experiment, the ambient temperature was strictly maintained at 25 °C, and the relative humidity was kept at 40 ± 5%.

### Antibacterial Activity

Slices of T_v_‐NC_500_@F evaporator surfaces, each 1 mm thick and 0.25 cm^2^ in area, were placed in a 96‐well plate. Each well was inoculated with 300 µL of a suspension containing 1 × 10^5^ CFU mL^−1^ of *S. aureus* and *E. Coli*. The experimental group (with photothermal material) was exposed to simulated sunlight for durations of 0, 30, and 60 min, while the control group (without photothermal material) was kept in darkness. After exposure, 50 µL of the bacterial suspension was uniformly spread onto LB agar plates and incubated at 37 °C for 24 h.^[^ ^13^, ^14^ ^]^ Colony counts were taken, and the experiment was repeated three times to ensure reproducibility.

### Organic Pollutant Degradation

Slices of T_v_‐NC_500_@F evaporator surfaces, each 1 mm thick and 0.25 cm^2^ in area, were placed in 100 mL of methylene blue (MB) solution and left to reach adsorption equilibrium for 60 min. The degradation reaction was then carried out under simulated sunlight, with 2 mL of solution sampled every 20 min to measure the absorbance and assess the degradation performance. Controlled experiments were conducted to determine which active species played a key role in the degradation of MB. Various scavengers were added:^[^ ^43^, ^44^ ^]^ 24 mL of isopropanol to capture hydroxyl radicals, 20 mg of benzoquinone to capture superoxide anions, 20 mg of potassium oxalate to capture holes, and 20 mg of silver nitrate to capture electrons. Every 20 min, 2 mL of the solution was sampled to measure absorbance and determine the contributions of different reactive species to the degradation process.

### Characterizations

Several characterizations were conducted in this study. The morphology and microstructure of the samples were observed using a scanning electron microscope (SEM, SU8010) and a transmission electron microscope (TEM, Tecnai G2 F20).^[^ ^45^, ^46^ ^]^ The surface element composition was analyzed using X‐ray photoelectron spectroscopy (XPS, ESCALAB 250Xi spectrometer, Thermo Scientific, USA) equipped with a monochromatic Al Ka X‐ray source. The results were calibrated with the C 1s signal set to 284.8 eV.^[^ ^47^ ^]^ The water contact angle was measured using a micro‐optical contact angle measurement system (ShengDing SDC). The absorption spectrum was recorded using a UV–vis‐NIR spectrophotometer (Lambda 750 S, PerkinElmer, USA).

### Calculation Details**—**Density Functional Theory (DFT) calculations

The calculations were performed using density functional theory (DFT) with the plane‐wave CASTEP code. The generalized gradient approximation (GGA) and PBE functional were employed.^[^ ^48^ ^]^ The cutoff energy was set to 450 eV. During the geometry optimization process, all atoms were allowed to relax without any constraints until the convergence thresholds for maximum displacement (1.0 × 10⁻^3^ Å), maximum force (0.03 eV Å^−1^), and energy (1.0 × 10⁻⁵ eV atom^−1^) were reached.^[^ ^49^ ^]^ Modeling of the nitrogen‐doped titanium dioxide with oxygen vacancies (T_v_‐NC_500_) material was performed by substituting nitrogen on the graphene surface and introducing oxygen vacancies in titanium dioxide.

### Calculation Details**—**Finite Element Simulation

Finite element simulations were conducted using the commercial finite element software package COMSOL Multiphysics 5.6. Initially, numerical simulations were employed to calculate the coordinates of regular dodecagons, regular pentagrams, regular dodecagrams, and skew dodecagrams, each with a base area of 4 cm^2^. Using these coordinates as the base, cylindrical evaporators were constructed. The side height of the evaporators was set to 6 cm, with photothermal materials coated on both the top and side surfaces. The simulations involved multiple physical fields,^[^ ^50^, ^51^ ^]^ as detailed model parameters can be found in the Supporting Information.

## Conflict of Interest

The authors declare no conflict of interest.

## Supporting information



Supporting Information

## Data Availability

The data that support the findings of this study are available from the corresponding author upon reasonable request.
