# Supplementary material for: Solar‐Driven Evaporator With “Starburst Turbine” Design Featuring Directional Salt Crystallization, Antibacterial, and Catalytic Multifunctionality for Efficient Water Purification
Source: Adv Sci (Weinh). 2024 Sep 25;11(43):2406696. doi: 10.1002/advs.202406696 (PMC11578366; doi:10.1002/advs.202406696)
Supplement: Supplementary file 1 — Supporting Information [file ADVS-11-2406696-s001.docx]

Solar-Driven Evaporator with “Starburst Turbine” Design Featuring Directional Salt Crystallization, Antibacterial, and Catalytic Multifunctionality for Efficient Water Purification

Jiahui Yu, Lele Li, Yuxuan Liu, Jie Wen, Shu Liu, Jiye Li, Junyi Ning, Changxiang Shao, Tao Wu*, Bing Liu*

J. Yu, L. Li, Y. Liu, J. Wen, J. Li, J. Ning, C.Shao, B.Liu

Department of Stomatology

Shandong Provincial Hospital Affiliated to Shandong First Medical University

Jinan 250021, China

E-mail: liubing@sdfmu.edu.cn

J. Yu, L. Li, Y. Liu, J. Wen, J. Li, J. Ning, C.Shao, B.Liu

Medical Science and Technology Innovation Center

School of Stomatology

Shandong First Medical University & Shandong Academy of Medical Sciences

Jinan 250117, China

T. Wu

Department of Chemical and Environmental Engineering

The University of Nottingham Ningbo China

Ningbo 315100, China

E-mail: tao.wu@nottingham.edu.cn

S.Liu

Nottingham Ningbo China Beacons of Excellence Research and Innovation Institute

University of Nottingham Ningbo China

Ningbo 315100, China

***Solar-to-vapor Conversion Efficiency***
Under steady-state conditions, the solar-thermal conversion efficiency (η_svc_) of the evaporator, a parameter extensively utilized in previous studies, is calculated as the ratio of the power consumed for water evaporation (Q_evap_) to the solar illumination power (Q_s_) ^[1-3]^.
 $\eta_{svc}=\frac{Q_{evap}}{Q_{s}}=\frac{ṁH_{s}}{C_{opt}P_{0}}$ Equation S1

$\dot{m}=m_{light}-m_{dark}$ Equation S2

In the expression, 𝑚̇ represents the net evaporation rate, which is derived after deducting the evaporation rate observed in a dark environment. H_s_ denotes the total enthalpy that encompasses both the sensible heat and the phase transition from liquid to vapor (J g^-1^). Additionally, C_opt​_ stands for the optical concentration, while P_0_​ is the standard direct solar illumination, quantified as 1 kW m^-2^.

To ascertain the equivalent evaporation enthalpy of the prepared evaporators (H_s_), a series of controlled comparative experiments were undertaken in a dark setting to maintain a uniform energy input (𝑈_𝑖𝑛_). The experimental setup comprised a closed vessel housing both the water and the constructed evaporator (2D), which were characterized by identical evaporation surface areas. The equivalent evaporation enthalpy (Hs) of the evaporator is estimated by the following equation ^[1-3]^:

$U_{in}=H_{vap}m_{0}=H_{s}m_{g}$ Equation S3
Where H_vap_ denotes the evaporation enthalpy of the bulk water, while 𝑚_0_ represents the associated mass change. Additionally, 𝑚_g_ is indicative of the mass change of the prepared evaporator.

Figure S2 presents the calculated Hs values for different materials. By substituting Hs into Equation S1, η can be obtained.

**The price of the foam**

The market price of melamine is approximately 910 dollar per ton, equating to 0.91 dollar per kilogram. The density of melamine foam is 10 kg m^-3^, resulting in a cost of 9.1 dollar per cubic meter for melamine foam. The volume of foam required for one evaporator is 28 cm³; thus, the cost of the foam for an evaporator discussed in this paper is calculated to be $2.55 × 10^-4^. Overall, these costs are considered insignificant.

**The cost of the T_v_-NC_500_ photothermal material**

The cost is calculated based on market prices of 1 kg 2-NH_2_-phthalic acid ($0.7) and 1 L tetra-n-butyl titanate ($5.38), resulting in a cost of $0.0177 to produce 2.1 g of T_v_-NC_500_ photothermal material. Consequently, the cost per gram of the photothermal material is $0.0084. Given that each evaporator in this study requires 0.4 g of photothermal material, the cost of the photothermal material per evaporator amounts to $3.36 × 10^-3^.

**The cost of one T_v_-NC_500_@F evaporator**

The total cost of one T_v_-NC_500_@F evaporator in this study includes both the foam cost and the photothermal material cost, totaling $3.615 × 10^-3^. It is evident that the cost of the evaporator is not high and falls within an acceptable range.

**The spraying process**

The spraying process was carried out using a spray gun connected to a gas cylinder (with a nozzle size of 0.5 mm), employing nitrogen as the propellant gas, and the pressure was controlled via the spray gun's valve. The spray gun was positioned 2 cm away from the evaporator, and the quantity of photothermal material was calculated based on a density of 0.1 g cm^-2^ per base area for uniform spraying.

**The significance of antibacterial capability**
Antibacterial capability holds several key significances for interfacial solar seawater desalination evaporators:

1. Extending Device Lifespan

During seawater desalination, the surface of the device is frequently exposed to humid and warm environments, which are highly conducive to bacterial growth and reproduction. The accumulation of bacteria can lead to biofilm formation on the surface of the device, potentially causing material corrosion and degradation, thereby shortening the device's lifespan.^[2]^ A desalination device with antibacterial properties can effectively inhibit bacterial growth, preventing these issues and extending the device's operational life.

2. Improving Desalination Efficiency

Biofilm formation can cover the surface of photothermal materials, obstructing light absorption and water evaporation, thus reducing the efficiency of the solar evaporator.^[3]^ Antibacterial properties can prevent biofilm formation, ensuring that the surface of the photothermal material remains clean, thereby maintaining high light absorption efficiency and evaporation rates, and ultimately enhancing overall desalination efficiency.

3. Ensuring Water Quality

During the seawater desalination process, excessive bacterial proliferation on the device surface may lead to bacterial contamination of the desalinated water, compromising the safety and quality of the output.^[4]^ A device with antibacterial capabilities can effectively kill or inhibit bacteria, ensuring that the desalinated water is free from harmful microorganisms, thus providing safe, potable water.

4. Reducing Maintenance Costs

Since antibacterial properties can prevent biofouling and material degradation, the frequency and complexity of device maintenance will be significantly reduced.^[3]^ This means that users do not need to clean or replace materials as frequently, thereby lowering maintenance costs and labor input, enhancing the device's economic and practical viability.

**Comprehensive Information in the COMSOL Model**

This system involves a photothermal layer at the top that absorbs solar radiation, generating high temperatures. This heat is transferred to a foam layer, composed of porous materials and liquid water, thereby producing steam. The capillary action of the porous materials in the foam layer ensures the influx of liquid water, replenishing water losses caused by evaporation. Simultaneously, ambient air flows tangentially across the top of the dual-layer system, carrying away water vapor via natural convection. The entire evaporation process encompasses three distinct physical processes, which are, heat transfer, vapor transport, and water transport. To analyze such a complex physical system, we have made the following assumptions^[5, 6]^:

For the photothermal layer:

(1) The thickness of the photothermal layer material is disregarded and it is considered as a diffuse reflector.

(2) Radiation heat losses from the photothermal layer to the environment are ignored.

For the foam layer:

(3) Radiation heat losses from the foam layer to the environment are ignored;

(4) The foam layer's porous materials exhibit isotropic properties.

(5) Viscous dissipation due to heat transport and pressure variations is neglected to satisfy the local thermal equilibrium assumption.

(6) During evaporation, the vapor and liquid water within the foam layer maintain a dynamic balance, i.e., the timescale of evaporation is significantly smaller than the timescales of heat and mass transfer.

(7) The system only absorbs liquid water from the bottom of the dual-layer structure.

For ambient air:

(8) Natural convection of ambient air is replaced by laminar flow of air.

For bulk water:

(9) Given that the system represents a small-scale evaporator, only the heat conduction capability of the bulk water at the bottom is considered. Convective heat transfer ability is disregarded, meaning the bulk water is considered static.

**1. Heat transfer**

1.1 Fourier's Law of Heat Conduction

This law is used to describe how heat propagates along a temperature gradient.

$q=-kA(\frac{dT}{dx})$ Equation S4

Here,

- q is the heat flux density (i.e., the heat flux per unit area per unit time), also known as the heat vector,

- k is the thermal conductivity of the material,

- A is the area through which the heat passes,

- dT/dx is the temperature gradient (i.e., the rate of change of temperature with location).

1.2 Energy Conservation Equation

This equation describes the relationship between temperature changes in a fluid and factors such as heat conduction, volume forces, viscous losses, and other heat sources. Specifically, the meanings of the terms in the equation are as follows:

$\rho vC_{p}\cdot\nabla T+\nabla\cdot q=Q+Q_{P}+Q_{vd}$ Equation S5

- ρ is the density of the fluid,

- v is the velocity vector of the fluid,

- C_p_ is the specific heat capacity of the fluid, measured in J/(kg·K), describing the temperature change of the fluid when absorbing or releasing heat,

- ∇ is the gradient operator,

- T is the temperature of the fluid,

- q is the heat conduction vector, describing the process of heat conduction from hot regions to cold regions within the fluid,

- Q is the external heat source,

- Q_p_ is the heat source produced by volume forces, such as the heat produced by the compression or expansion of the fluid,

- Q_vd_ is the heat source produced by viscous losses in the fluid, where the frictional force generated by fluid viscosity is converted into heat internally.

**2. Laminar Flow**

2.1 Continuity Equation

$\nabla\cdot(\rho v)=0$ Equation S6

This formula applies to incompressible fluids.

- ρ is the density of the fluid,

- v is the velocity vector of the fluid,

- ∇• is the divergence operator.

2.2 Momentum Conservation Equation

$\rho\left( v\cdot\nabla\right)v=-\nabla\cdot\left[ pl+K \right]+F$ Equation S7

- ρ is the density of the fluid,

- v is the velocity vector of the fluid,

- ∇ is the gradient operator,

- p is pressure,

- I is the identity matrix,

- K is the stress tensor, which describes shear stress and dilatational stress within the fluid,

- F represents volume forces, such as gravity or electromagnetic forces, etc.

**3. Mass Transfer**

3.1 Fick's laws of diffusion

For steady-state diffusion, the diffusion flux of a substance is directly proportional to the concentration gradient:

$J=-D\cdot(\frac{\partial C}{\partial X})$ Equation S8

- J is the diffusion flux of the substance, representing the amount of substance passing through a unit area per unit time,

-D is the diffusion coefficient or diffusion constant, which is a physical property parameter measuring the ability of a substance to diffuse,

- ∂C/∂x is the concentration gradient.

The negative sign indicates that diffusion always proceeds from regions of high concentration to regions of low concentration.

The equation provides the general definition of evaporative efficiency:

$\eta=(\Delta m\cdot H_{tot})/q_{e}$ Equation S9

-Δm represents the change in mass of the bulk water,

-H_tot_ represents the total enthalpy of the phase change (including sensible heat and latent heat of vaporization),

-q_e_ represents the solar radiation heat flux.


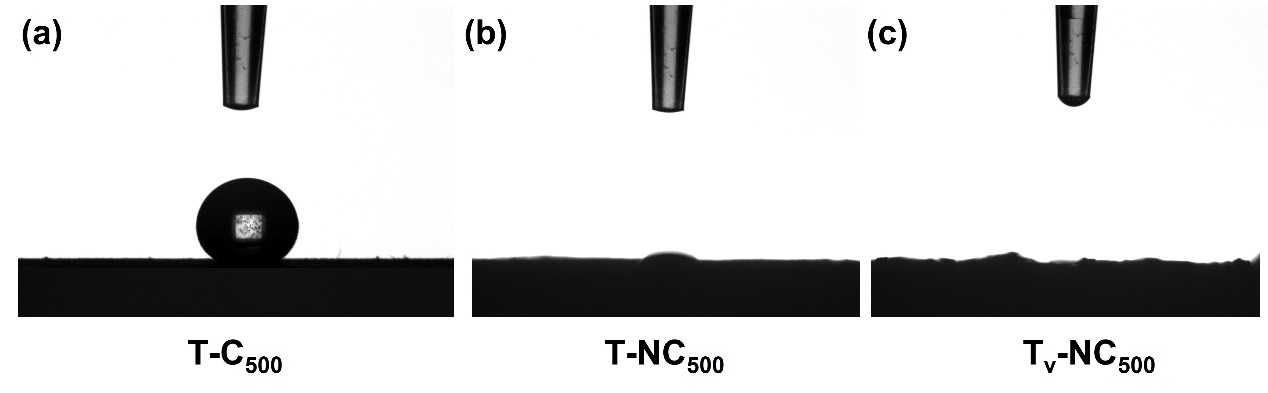


Figure S1 Contact Angle Tests of (a) T-C_500_, (b)T-NC_500_, (c)T_v_-NC_500_.

Photographs of contact angle were taken 3 seconds after the droplet was applied. In Figure S1(a), it can be seen that the T-C_500_ material exhibits poor hydrophilicity. In contrast, the nitrogen-doped T-NC_500_ and T_v_-NC_500_ materials show excellent hydrophilicity. This indicates that nitrogen doping significantly improves the hydrophilic properties of the photothermal materials.


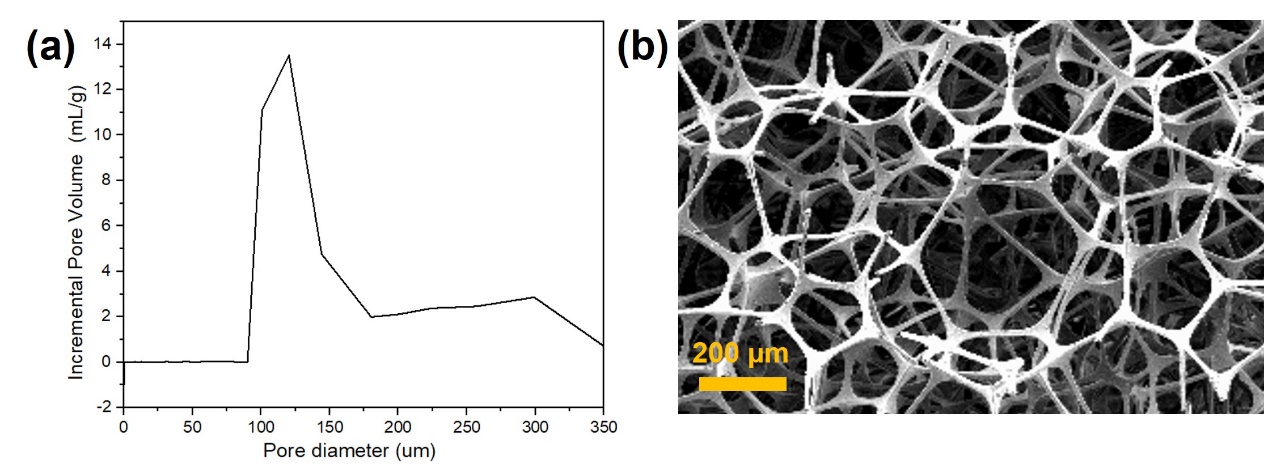
Figure S2 (a) Pore size distribution of Melamine Foam, (b)SEM image of Melamine Foam.

From Figure S2, it can be observed that the pore size of Melamine Foam ranges between 100-150 nm, with a porosity of 98.4 % (Based on the results obtained from mercury intrusion porosimetry).


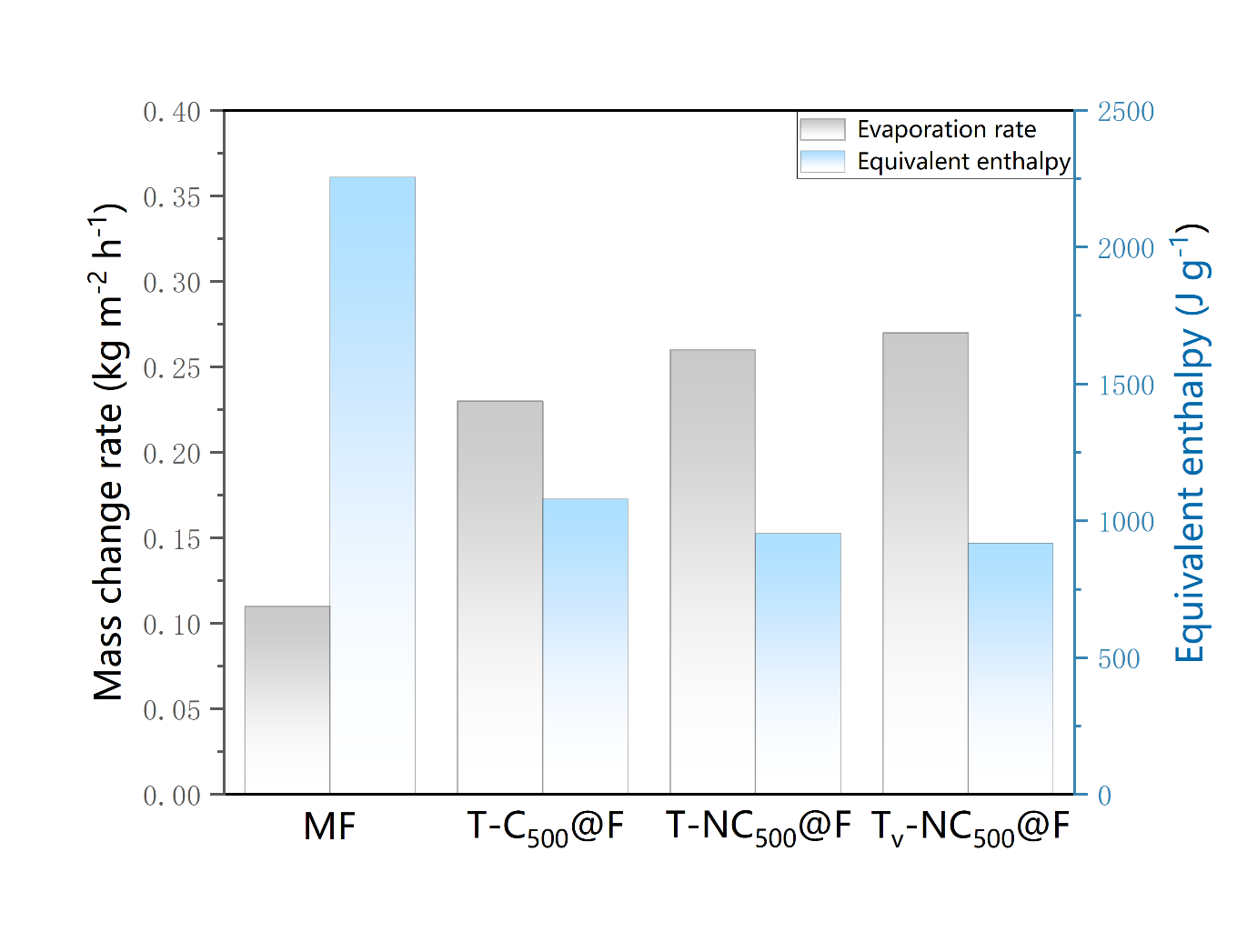


Figure S3 The Mass change rate (m_g_) and equivalent enthalpy of bulk water, T-C_500_@F, T-NC_500_@F and T_v_-NC_500_@F.^[4]^


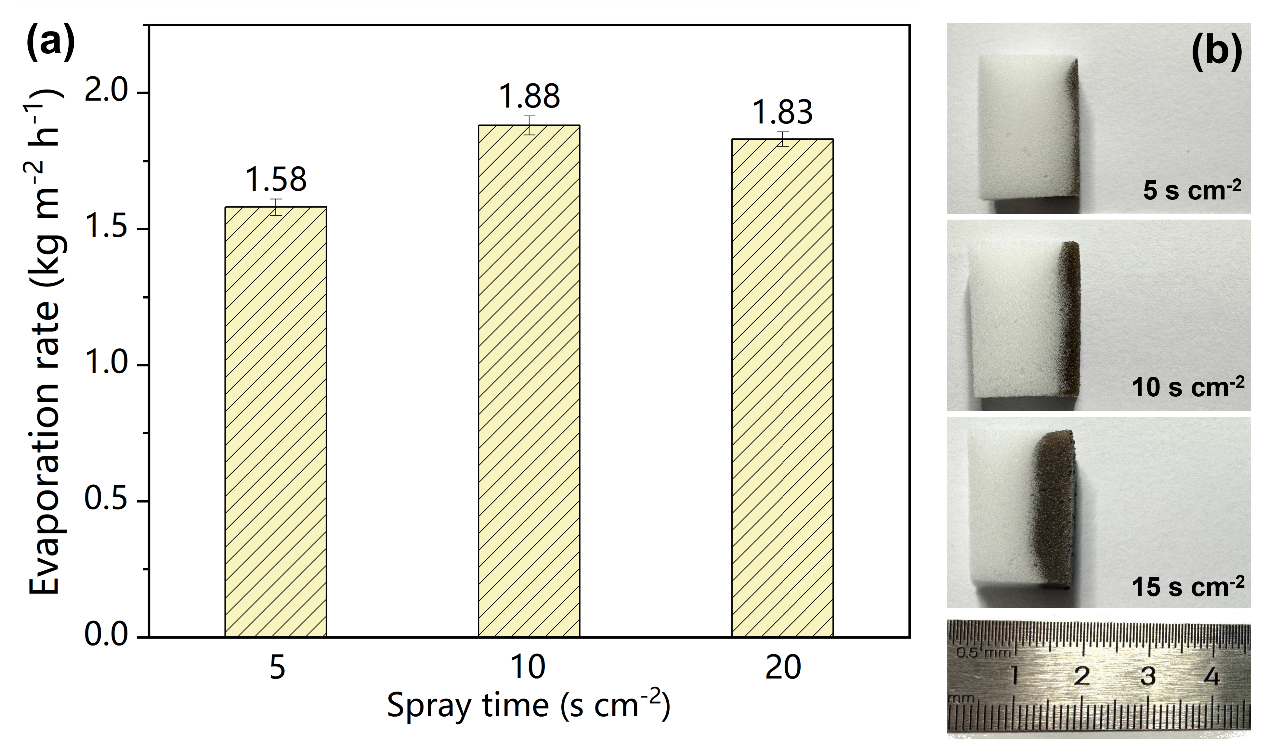


Figure S4 (a) Evaporation rates of evaporators formed with different spraying durations; (b) thickness comparison of these evaporators.

By spraying T_v_-NC_500_ onto flat 2D melamine foam, the thickness of the photothermal material can be controlled by adjusting the spraying duration to 5s cm⁻², 10s cm⁻², and 20s cm⁻² under the same spray gun pressure and dispersant concentration (0.1g ml⁻¹). This results in evaporators with photothermal layer thicknesses of 1mm, 3mm, and 5.5mm, respectively (as shown in Figure S4(b)). The evaporation performance of 2D evaporators with different spraying thicknesses is shown in Figure S4(a). It can be observed that the 2D evaporator formed with a 10s cm⁻² spraying duration exhibits the best evaporation performance, reaching 1.88 kg m⁻² h⁻¹. However, as the spraying thickness further increases, the evaporation rate decreases to 1.83 kg m⁻² h⁻¹, likely due to the excessive thickness of the photothermal layer hindering water transport.


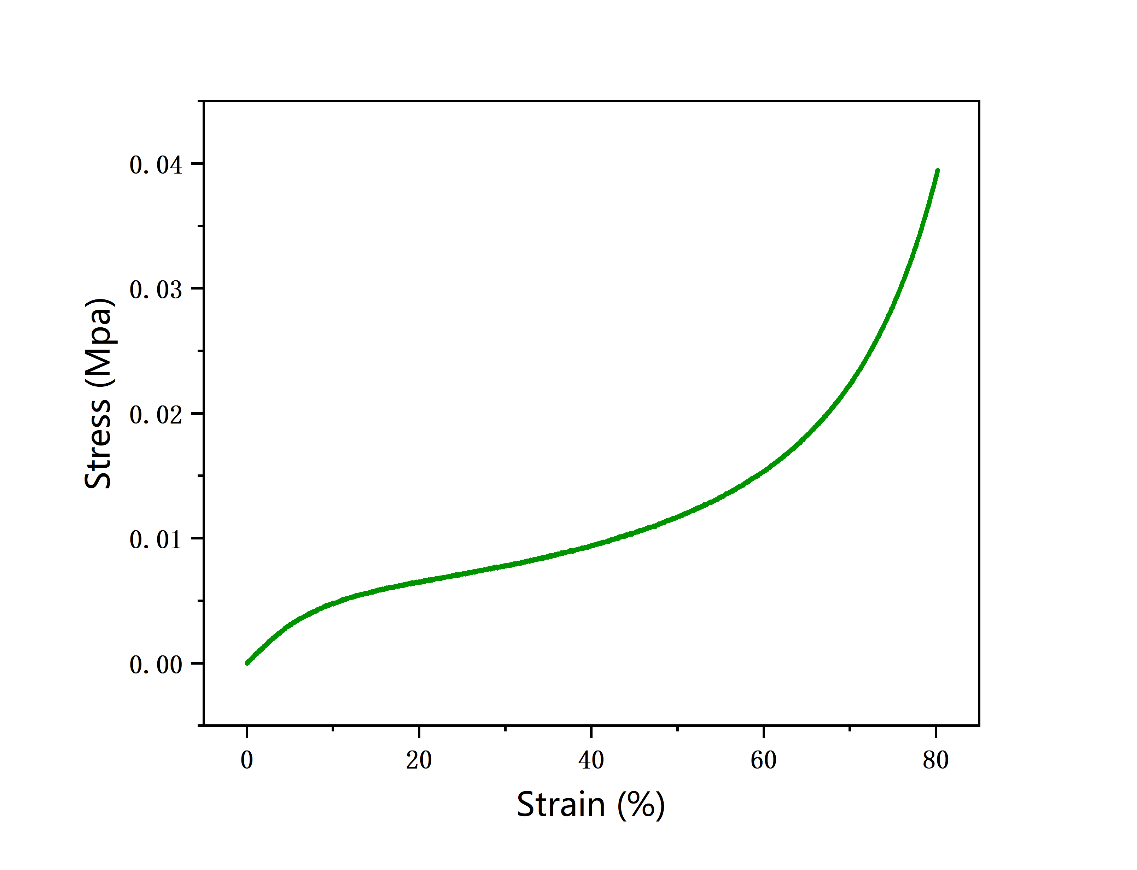
 Figure S5 Stress-Strain Curve of the T_v_-C_500_@F Evaporator.

As shown in Figure S5, it can be observed that as the strain increases, the stress also increases, demonstrating the nonlinear characteristics of the material. Initially, the relationship between stress and strain is relatively gentle, indicating lower stiffness in the small strain range. As the strain increases, the rate of stress increase accelerates, and the curve becomes steeper, suggesting that the material exhibits higher stiffness and strength at greater strains. Overall, the evaporator exhibits certain elastoplastic characteristics, being able to withstand gradually increasing compressive stress over a wide range of deformation. Thank you for your inquiry; the relevant content has been added to the Supporting Information and the revised manuscripts.


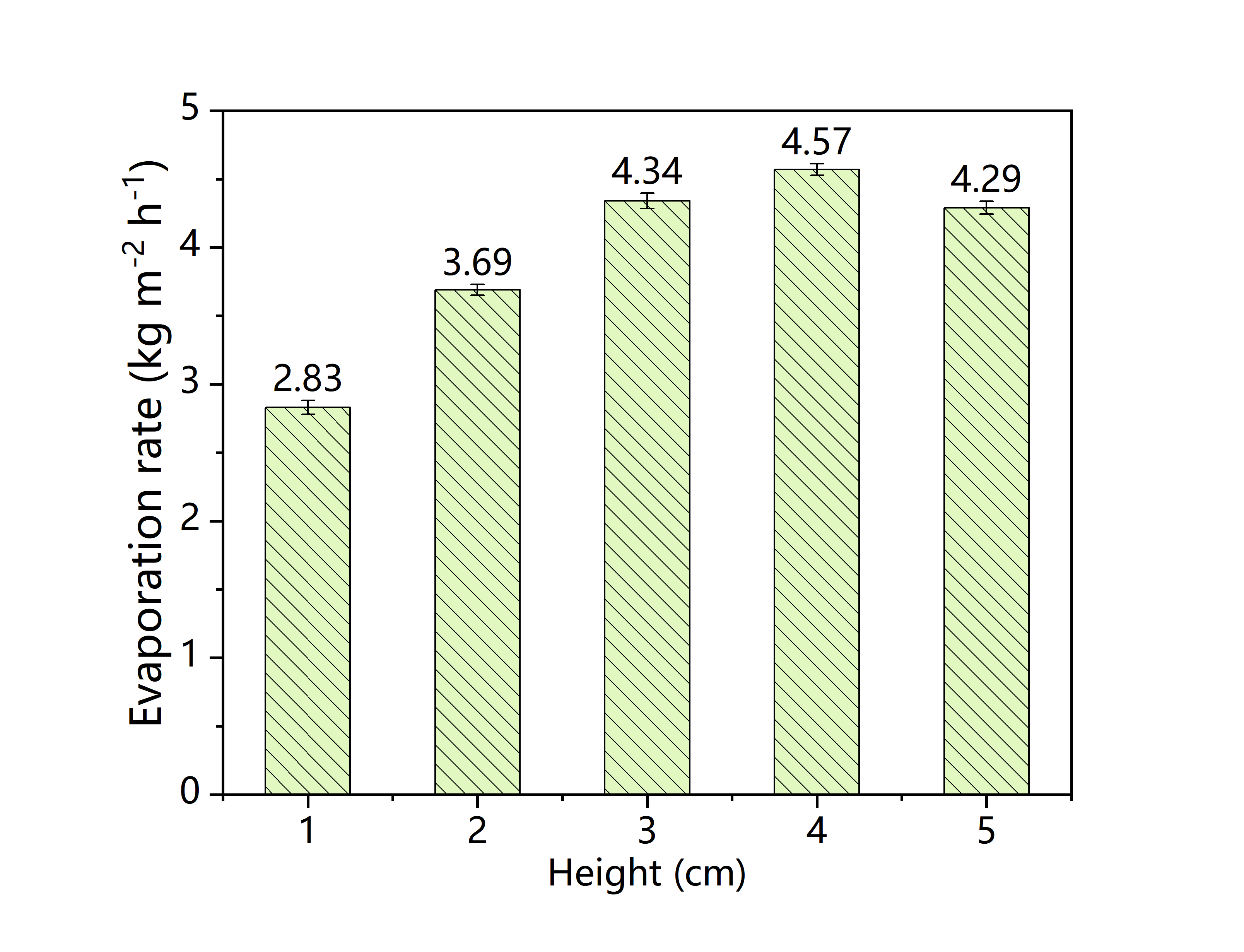
Figure S6: Evaporation performance of T_v_-NC_500_@F evaporators with different heights.

Figure S6 provides the evaporation performance of evaporators with different heights. It can be observed that initially, the evaporation efficiency increases with the height of the T_v_-NC_500_@F evaporator, reaching a peak at a height of 4 cm (4.57 kg m⁻² h⁻¹). However, the evaporation rate then begins to decline due to insufficient water supply. Therefore, the evaporator height selected in the main text is 4 cm.


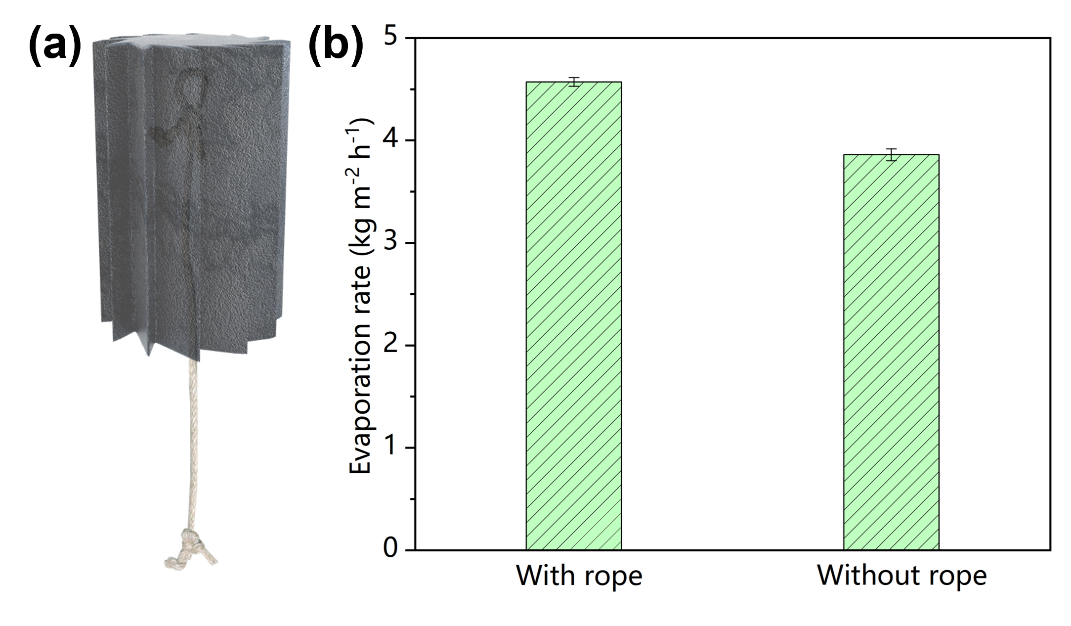
Figure S7: (a) Schematic diagram of the evaporator and absorbent cotton wicks; (b) The effect of adding absorbent cotton wicks on evaporation performance.


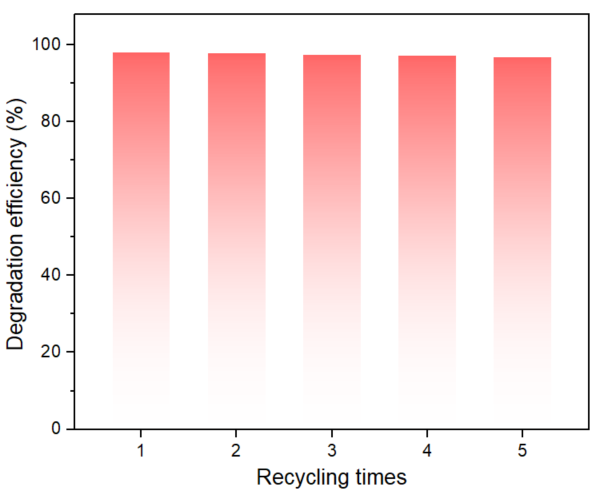
Figure S8 the reusability of T_v_-NC_500_@F for the photodegradation.


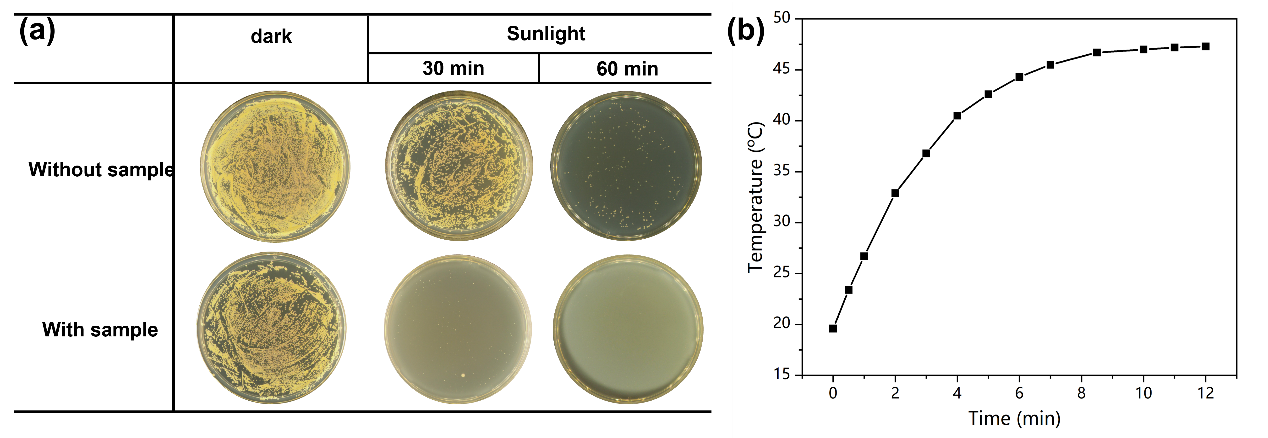


Figure S9: (a) Antibacterial performance of Petri dishes with and without samples at the same temperature; (b) Surface temperature of the evaporator during the antibacterial test.

Figure S9(b) shows the changes in the surface temperature of the evaporator. It can be observed that the temperature stabilizes after 10 minutes of illumination and remains around 47.2°C. Therefore, the control group (without sample) was placed in an incubator at 47.2°C to match the temperature of the experimental group, and its antibacterial performance against Staphylococcus aureus was tested. As shown in Figure R3(a), the Petri dish without the sample still had a relatively high number of bacteria after 30 minutes of incubation at 47.2°C; however, the bacterial count significantly decreased after 60 minutes, indicating that this temperature has a certain degree of bactericidal ability. For the Petri dish with the sample under illumination, the bacterial count significantly decreased after 30 minutes, and no bacteria were visible after 60 minutes, demonstrating that the ROS generated by the material under illumination is the main reason for its excellent antibacterial capability, with the temperature increase under illumination providing additional assistance in sterilization.


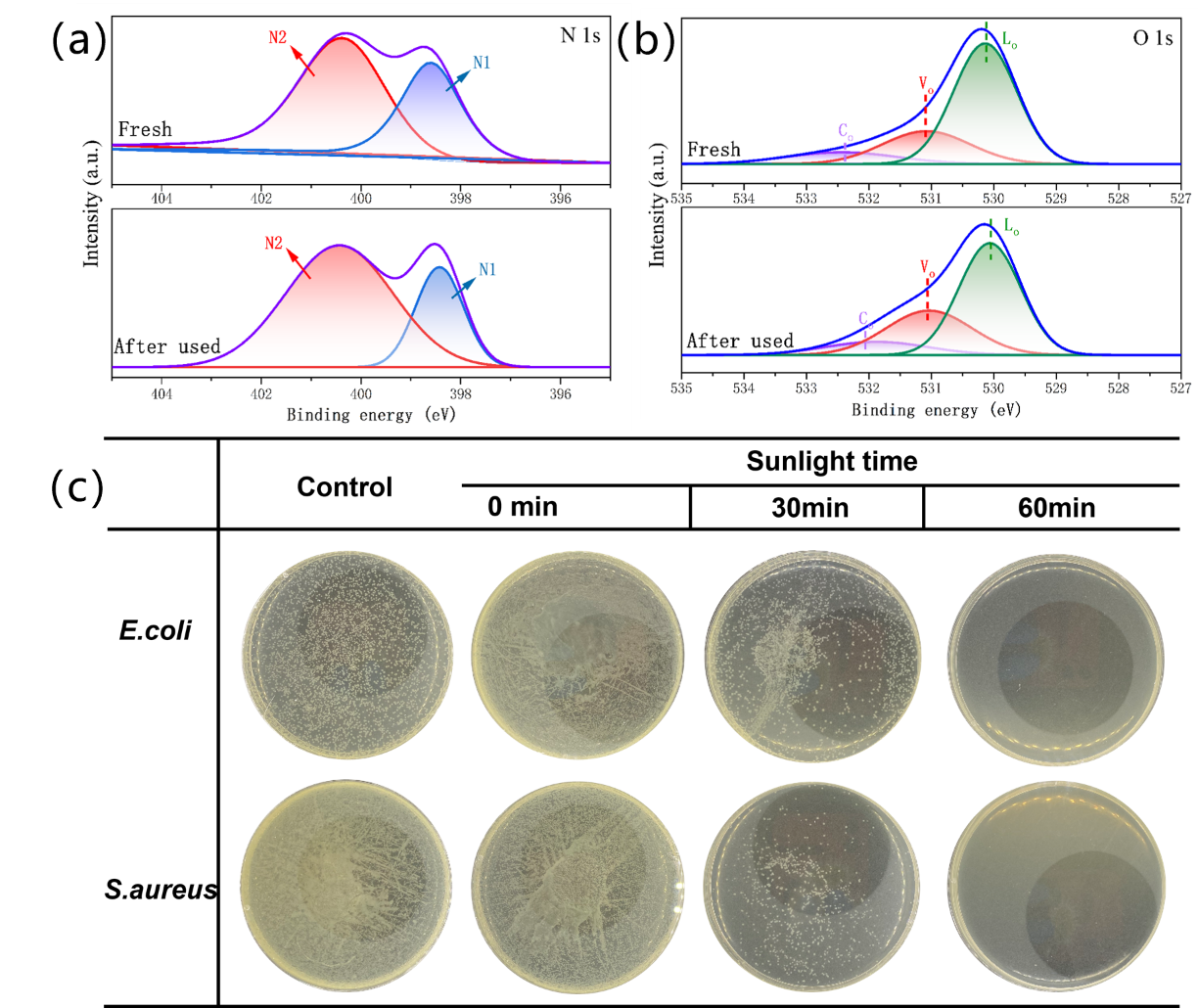


Figure S10: XPS results of T_v_-NC_500_ after 5 days of continuous testing (a) N 1s, (b) O 1s orbitals; and (c) antibacterial performance test of the material after 5 days of continuous testing.

The photothermal material from the evaporator after 5 days of continuous testing was removed and subjected to XPS analysis. The test results, as shown in Figure S10(a, b), indicate that after 5 days of continuous testing, there was no significant difference in the nitrogen doping content and oxygen vacancy (V_o_) content in the photothermal material, demonstrating that the photothermal material itself has good temporal stability. Additionally, the antibacterial performance of the photothermal material after 5 days of testing was tested. It was observed that after 30 minutes of illumination, the bacterial count significantly decreased, and after 60 minutes of illumination, 100% sterilization was achieved. This performance is consistent with the antibacterial properties of the unused T_v_-NC_500_ (as shown in Figure 4(d)), indicating that the material maintains its excellent antibacterial capability even after continuous operation, which is significant for the sustained stable operation and practical application of the evaporator.


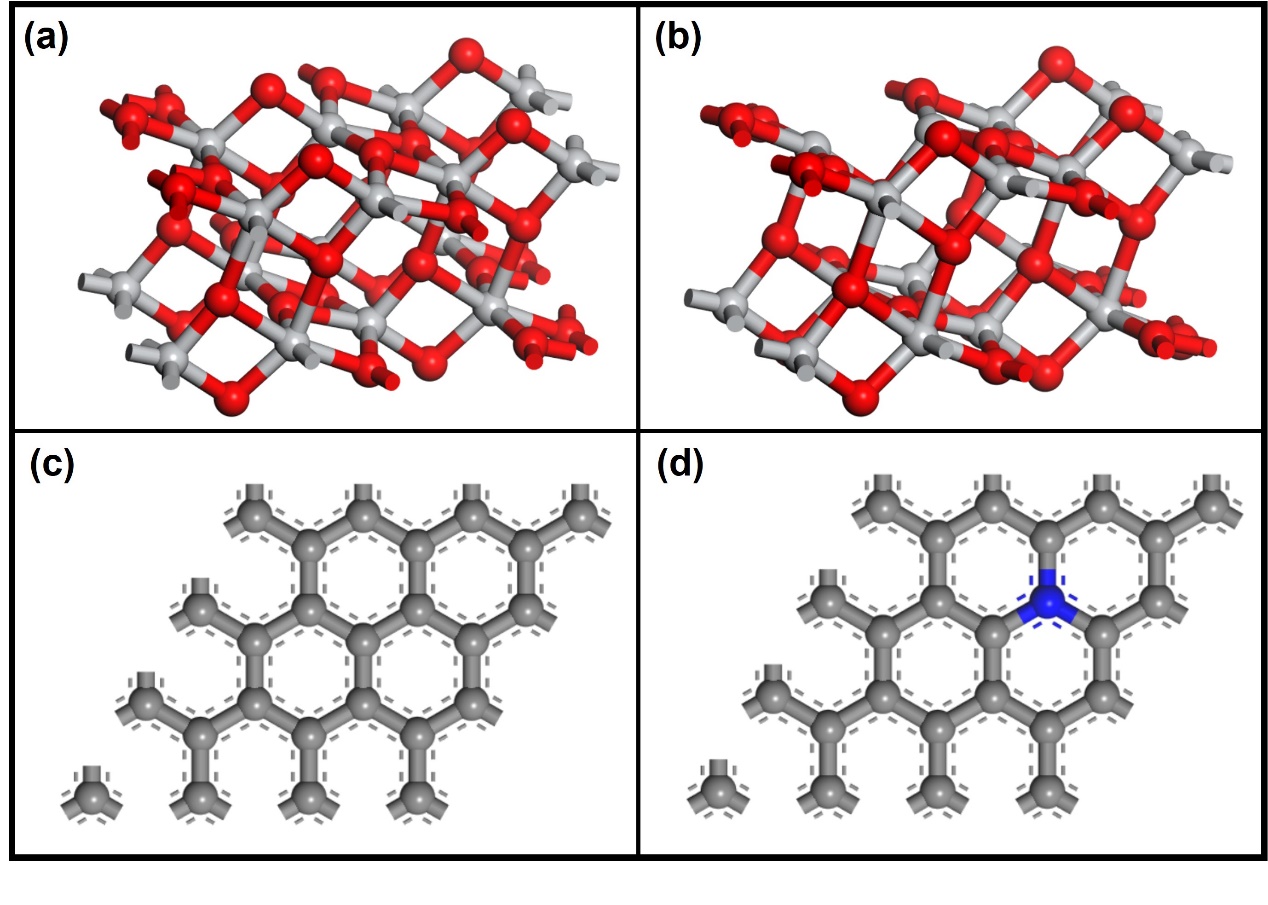
Figure S11 (a) TiO_2_; (b) TiO_2_ with oxygen vacancies; (c) graphene; (d) nitrogen-doped graphene.


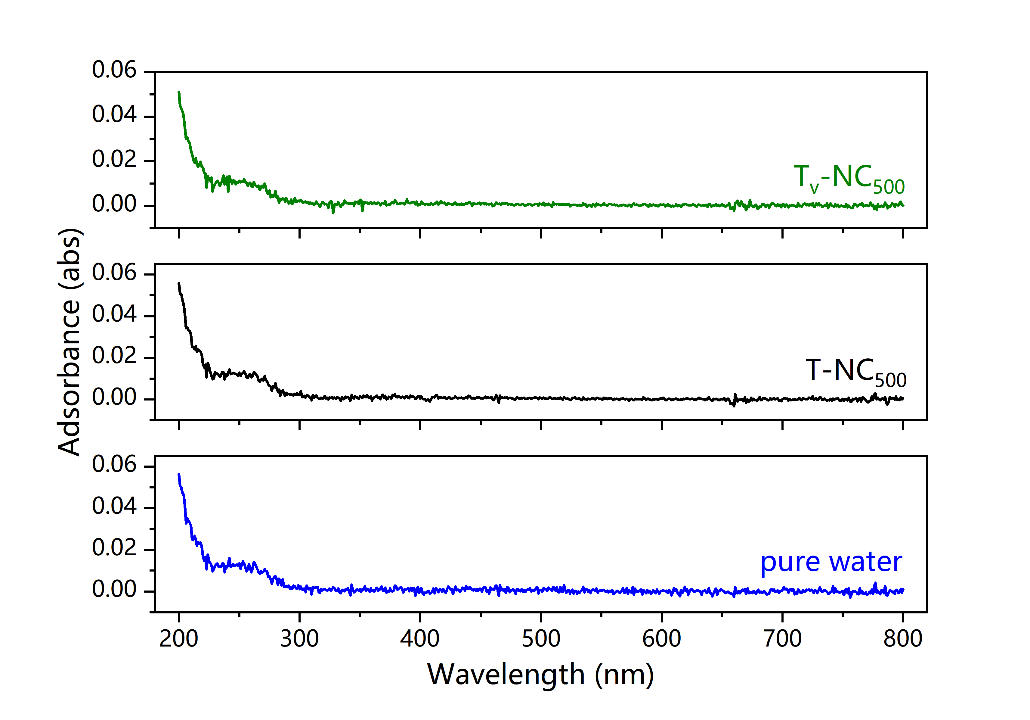
Figure S12 The absorbance data of water samples after soaking T_v_-NC_500_@F and T-NC_500_@F for five days, along with that of pure water.

As shown in Figure R8, the absorbance of pure water is not different from that of the water samples after being soaked with T_v_-NC_500_@F and T-NC_500_@F for five days. No peaks with absorbance values greater than 0.1 are observed in the figure, with 0.1 being a common threshold for potential contamination. This indicates that the water quality does not show any signs of contamination after the materials were soaked.

Furthermore, COD and ICP-MS were also used to monitor whether the water was contaminated after soaking with T_v_-NC_500_@F and T-NC_500_@F. According to the "Standards for Drinking Water Quality" (GB 5749-2006), the COD in drinking water should not exceed 3 mg L^-1^. The COD values of the water samples after soaking the T_v_-NC_500_@F evaporator and T-NC_500_@F evaporator for five days were 1.6 mg L^-1^ and 2.7 mg L^-1^, respectively, which meet the drinking water standards. ICP-MS was used to monitor the titanium content in the water, and the titanium ion concentrations in the water samples after soaking the T_v_-NC_500_@F evaporator and T-NC_500_@F evaporator were 0.272 µg L^-1^ and 0.07 µg L^-1^, respectively. These concentrations are very low, indicating that the evaporators do not contaminate the water source.

In conclusion, based on the results of UV-vis, COD, and ICP-MS, it has been demonstrated that the pure water was not contaminated after soaking the evaporator for five days, indicating that the evaporator is safe.


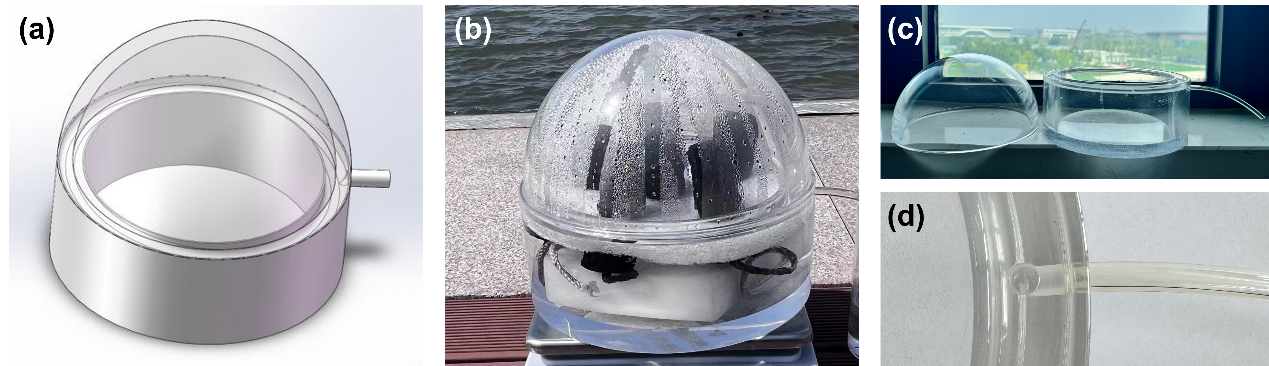


Figure S13 (a) the design diagram of the evaporation device; (b,c) photographs of the actual evaporation device; and (d) details of the groove.

In the design of the evaporation device, acrylic sheets with over 95% light transmittance were selected as the construction material. The device is planned to consist of two main components: the top cover and the base. The top cover is pre-designed as a hollow hemispherical shape, where water vapor condenses upon cooling. This shape allows the material to receive maximum sunlight exposure while also ensuring that the condensed water flows down smoothly. The base is generally shaped as a hollow cylinder with an opening at the top, serving as the main body for placing the material. A groove is set within the ring wall at the top of the base to collect the condensed water, with the lowest point of the groove drilled with a water outlet connected to a conduit (Figure S13(d)). This design facilitates the process of condensing and collecting the evaporated water vapor.
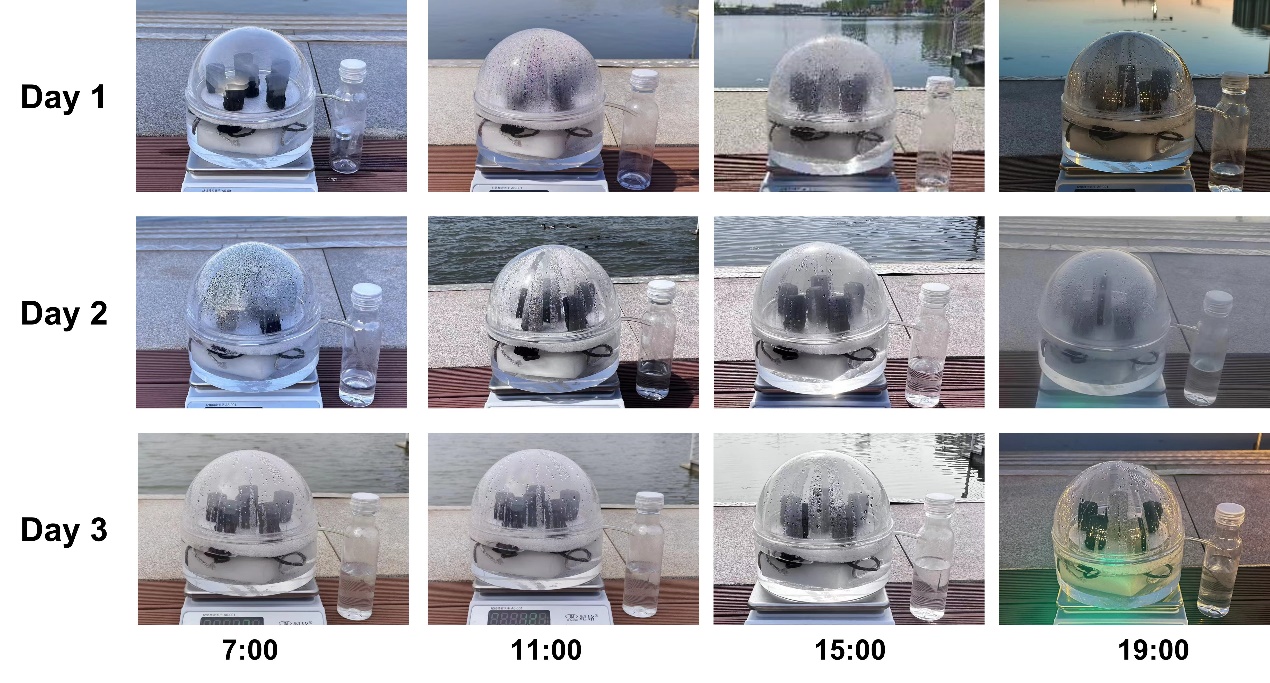
Figure S14 Photographs of the evaporation and water collection processes over three days for six T_v_-NC_500_@F evaporators.Table S1 Material properties and parameters used in the simulations

| **Parameters** | **Value** | **Unit** |
| --- | --- | --- |
| p_0_ | 1 | atm |
| T_0_ | 293.15 | K |
| D_va_ | 2.6*10^-5^ | m^2^ s^-1^ |
| H_evap_ | 2.454*10^6^ | J kg^-1^ |
| mu_a_ | 1.81*10^-5^ | kg m^-1^ s^-1^ |
| k_a_ | 0.025 | W m^-1^ K^-1^ |
| cp_a_ | 1.006*10^3^ | J kg^-1^ K^-1^ |
| rho_a_ | 1.205 | kg m^-3^ |
| Mn_l_ | 0.018 | kg mol^-1^ |
| mu_l_ | 1.002*10^-3^ | kg m^-1^ s^-1^ |
| k_l_ | 0.59 | W m^-1^ K^-1^ |
| cp_l_ | 4.182*10^3^ | J kg^-1^ K^-1^ |
| rho_l_ | 998.2 | Kg m^-3^ |
| cp_v_ | 2.062*10^3^ | J kg^-1^ K^-1^ |
| por | 0.984 | - |
| kappa | 1*10^-14^ | m^2^ |
| k_s_ | 0.14 | W m^-1^ K^-1^ |

**Reference**

[1] X. Liu, F. Chen, Y. Li, H. Jiang, D.D. Mishra, F. Yu, Z. Chen, C. Hu, Y. Chen, L. Qu, 3D Hydrogel Evaporator with Vertical Radiant Vessels Breaking the Trade‐Off between Thermal Localization and Salt Resistance for Solar Desalination of High‐Salinity, Advanced Materials, 34 (2022) 2203137.

[2] X. Zhou, Y. Guo, F. Zhao, W. Shi, G. Yu, Topology‐controlled hydration of polymer network in hydrogels for solar‐driven wastewater treatment, Advanced Materials, 32 (2020) 2007012.

[3] P. Liu, Y.b. Hu, X.Y. Li, L. Xu, C. Chen, B. Yuan, M.L. Fu, Enhanced Solar Evaporation Using a Scalable MoS2‐Based Hydrogel for Highly Efficient Solar Desalination, Angewandte Chemie, 134 (2022) e202208587.

[4] D. Wei, C. Wang, J. Zhang, H. Zhao, Y. Asakura, M. Eguchi, X. Xu, Y. Yamauchi, Water Activation in Solar‐Powered Vapor Generation, Adv. Mater., 35 (2023) 2212100.

[5] L. Li, N. He, B. Jiang, K. Yu, Q. Zhang, H. Zhang, D. Tang, Y. Song, Highly salt‐resistant 3D hydrogel evaporator for continuous solar desalination via localized crystallization, Advanced Functional Materials, 31 (2021) 2104380.

[6] S. Cheng, Y. Li, B. Jin, Z. Yu, R. Gu, Designing salt transmission channel of solar-driven multistage desalination device for efficient and stable freshwater production from seawater, Desalination, 531 (2022) 115688.
